# Chronic activation of hexosamine biosynthesis in the heart triggers pathological cardiac remodeling

**DOI:** 10.1038/s41467-020-15640-y

**Published:** 2020-04-14

**Authors:** Diem Hong Tran, Herman I. May, Qinfeng Li, Xiang Luo, Jian Huang, Guangyu Zhang, Erica Niewold, Xiaoding Wang, Thomas G. Gillette, Yingfeng Deng, Zhao V. Wang

**Affiliations:** 1 Division of Cardiology, Department of Internal Medicine, University of Texas Southwestern Medical Center, Dallas, TX USA; 2 Key Laboratory of Cardiovascular Intervention and Regenerative Medicine of Zhejiang Province, Department of Cardiology, Sir Run Run Shaw Hospital, School of Medicine, Zhejiang University, Hangzhou, Zhejiang China; 3 Touchstone Diabetes Center, Department of Internal Medicine, University of Texas Southwestern Medical Center, Dallas, TX USA

**Keywords:** Cell signalling, Metabolism, Cardiac hypertrophy

## Abstract

The hexosamine biosynthetic pathway (HBP) plays critical roles in nutrient sensing, stress response, and cell growth. However, its contribution to cardiac hypertrophic growth and heart failure remains incompletely understood. Here, we show that the HBP is induced in cardiomyocytes during hypertrophic growth. Overexpression of Gfat1 (glutamine:fructose-6-phosphate amidotransferase 1), the rate-limiting enzyme of HBP, promotes cardiomyocyte growth. On the other hand, Gfat1 inhibition significantly blunts phenylephrine-induced hypertrophic growth in cultured cardiomyocytes. Moreover, cardiac-specific overexpression of Gfat1 exacerbates pressure overload-induced cardiac hypertrophy, fibrosis, and cardiac dysfunction. Conversely, deletion of Gfat1 in cardiomyocytes attenuates pathological cardiac remodeling in response to pressure overload. Mechanistically, persistent upregulation of the HBP triggers decompensated hypertrophy through activation of mTOR while Gfat1 deficiency shows cardioprotection and a concomitant decrease in mTOR activity. Taken together, our results reveal that chronic upregulation of the HBP under hemodynamic stress induces pathological cardiac hypertrophy and heart failure through persistent activation of mTOR.

## Abstract (web summary)

Metabolic remodeling plays an important role in pathological cardiac hypertrophy. Here, the authors show that hexosamine biosynthetic pathway is elevated in the heart by pressure overload, which contributes to heart failure by persistent activation of mTOR.

## Introduction

Glucose metabolism plays an essential role in both physiology and pathophysiology. After entering the cells, glucose is catabolized via multiple pathways, including glycolysis, glycogen synthesis, the polyol pathway, pentose phosphate pathway, and hexosamine biosynthetic pathway (HBP). At baseline, the HBP utilizes approximately 2–5% of incoming glucose; however, contribution through this route can be significantly elevated under stress conditions^1^. The HBP flux is mainly governed by nutrient intake (glucose and glutamine) and the rate-limiting enzyme glutamine:fructose-6-phosphate amidotransferase (Gfat)^2,3^. Gfat converts fructose-6-phosphate and glutamine to glucosamine-6-phosphate (GlcN-6-P). The following reactions of the HBP then catalyze GlcN-6-P to uridine diphosphate N-acetylglucosamine (UDP-GlcNAc), an indispensible sugar nucleotide to synthesize glycosaminoglycan, proteoglycan, glycolipid, and O-linked GlcNAc protein modification (O-GlcNAcylation). Aberrant regulation of HBP has been implicated in various diseases, including neurodegeneration, cancer, and heart disease^4–8^. We recently showed that cardiac HBP and O-GlcNAc modification are strongly induced in ischemic heart disease^9^. Moreover, spliced X-box binding protein 1 (XBP1s), a key transducer of unfolded protein response (UPR), is a direct upstream transcriptional factor of multiple enzymes of the HBP, including Gfat1^9^. Suppression of Gfat1 and the HBP significantly diminishes XBP1s-mediated cardioprotection against ischemic heart disease^9^. Despite our ample understandings of the HBP in cardiac ischemia, its role in hypertensive heart disease remains elusive.

Hypertension is one of the most important risk factors for heart failure^10^, a condition currently affecting 6 million Americans^11^ with overwhelming healthcare and socioeconomic impact^12^. In response to hemodynamic stress, cardiac ventricles increase thickness to alleviate wall stress and the heart subsequently enlarges^13^. This hypertrophic growth initially aims to maintain heart function, which is considered as an adaptive feedback reaction. However, sustained stress induces the progression from compensation to maladaptation, and heart failure may ensue. Despite extensive interests and profound clinical relevance^14^, signaling pathways and pathological triggers of this transition are ill-defined. Numerous studies suggest that metabolic derangement is one of the most important and earliest processes underlying pathological cardiac remodeling in response to hypertension^15–19^. The normal heart preferentially uses fatty acids to produce ATP whereas in the hypertrophied heart, glucose consumption is elevated^20,21^. Indeed, cardiac hypertrophy is accompanied by a 50% increase in glucose uptake while glucose oxidation via the tricarboxylic acid cycle remains largely unchanged^22^. Intracellular glucose may therefore be shunted to other metabolic pathways, including the HBP. Consistent with this notion, elevation of cardiac UDP-GlcNAc has been discovered in rats by pressure overload^23^. Moreover, O-GlcNAc protein modification in hearts is increased in various models of cardiac hypertrophic growth^24^. More recently, Gelinas et al.^25^ showed that 5′-adenosine monophosphate-activated protein kinase (AMPK) prevents pathological cardiac hypertrophy by promoting Gfat1 phosphorylation and thereby decreasing O-GlcNAc modification in the heart. Despite these findings, it remains to be answered whether the upregulation of HBP by pressure overload plays a causal role in pathological cardiac remodeling.

Mechanistic/mammalian target of rapamycin (mTOR) is an atypical protein kinase, consisting of two distinct complexes to integrate multiple metabolic signals and govern cell growth^26^. Early studies showed that glucose infusion in ex vivo hearts leads to load-induced mTOR activation, which precedes the development of cardiac dysfunction^27^. On the other hand, glucose metabolism and the cardiac UDP-GlcNAc level are strongly elevated in hypertrophied hearts^23^. These findings suggest that the HBP might directly regulate mTOR signaling in pathological cardiac remodeling.

Here, we show that the HBP is significantly elevated in cardiomyocytes during hypertrophic growth. Cardiac-specific overexpression of Gfat1 potentiates pathological cardiac remodeling by pressure overload whereas inducible deletion of Gfat1 confers cardioprotection. Our data reveal that chronic upregulation of the HBP and consequent O-GlcNAcylation in the heart leads to persistent activation of mTOR, exacerbated pathological cardiac remodeling, and heart failure.

## Results

### The HBP is induced by hypertrophic growth in cardiomyocytes

**Fig. 1:**
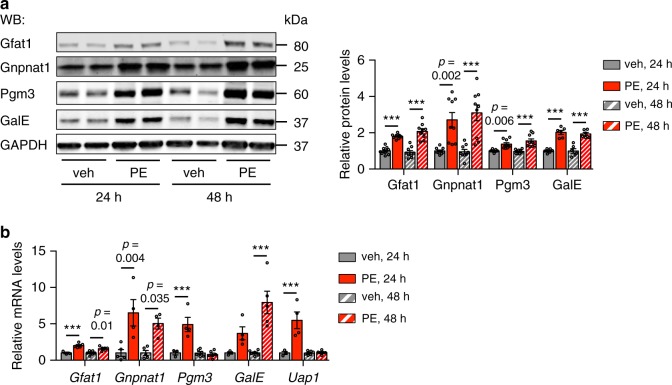
Induction of the hexosamine biosynthetic pathway in cardiomyocytes. **a** Neonatal rat ventricular myocytes (NRVMs) were treated with phenylephrine (PE, 50 μM) for 24 and 48 h, respectively. Representative immunoblots of the hexosamine biosynthetic pathway (HBP) enzymes (Gfat1, Gnpnat1, Pgm3, and GalE) are shown. Quantification (right) indicates significant upregulation of the HBP by PE treatment in cultured cardiomyocytes. *N* = 6 for GalE quantification and *n* = 9 for all other proteins. **b** PE treatment led to strong increases of the HBP enzymes at the mRNA level. *N* = 3–6. Data are shown as mean ± SEM. Significance was calculated by two-way ANOVA, followed by Tukey’s test. ***p < 0.001. Source data are provided as a Source Data file.

The HBP is a series of enzymatic reactions to convert glucose to UDP-GlcNAc (Supplementary Fig. 1a). We first determined whether the HBP enzymes were altered in cultured cardiomyocytes by hypertrophic stimuli in a time course study. Phenylephrine (PE) is an agonist for α1 adrenergic receptor, which is commonly used to stimulate cardiomyocyte hypertrophic growth in vitro^28–30^. We isolated and cultured primary neonatal rat ventricular myocytes (NRVMs) from 1 to 2 days old Sprague–Dawley rats as before^9,31^. We treated NRVMs with PE (50 μM) to induce hypertrophy for 24 or 48 h. PE promoted a significant increase of cardiomyocyte size as shown by α-actinin immunostaining and quantification (Supplementary Fig. 1b). Consistently, cardiomyocyte protein synthesis was augmented as revealed by a leucine incorporation assay (Supplementary Fig. 1c). Moreover, the mRNA level of genes of the fetal program as molecular markers of hypertrophic growth (e.g., *Anf*, *Bnp*, and *βMHC*) was greatly upregulated (Supplementary Fig. 1d). Notably, the magnitude of hypertrophic response at 48 h was significantly stronger than 24 h (Supplementary Fig. 1b–d). Importantly, we found that the protein levels of the HBP enzymes Gfat1, Gnpnat1, Pgm3, and GalE were significantly elevated (Fig. 1a), consistent with the mRNA upregulation (Fig. 1b). We next examined whether other hypertrophic stimuli might similarly induce the HBP. Treatment by IGF-1, Endothelin-1 (ET-1), or Angiotensin II (Ang II) in NRVMs led to hypertrophic growth and concomitant increases in genes of the HBP (Supplementary Fig. 2a–c). Although NRVMs resemble many characteristics of adult cardiomyocytes, significant differences exist. To further evaluate the induction of the HBP by hypertrophic growth, we isolated mouse adult cardiomyocytes and treated with PE. Interestingly, PE-induced hypertrophic growth in adult cardiomyocytes (Supplementary Fig. 3a) was also associated with a significant increase of Gfat1 expression (Supplementary Fig. 3b). Taken together, these findings suggest that cardiomyocyte hypertrophic growth is associated with significant upregulation of the HBP.

### Overexpression of Gfat1 induces hypertrophic growth in cardiomyocytes

**Fig. 2:**
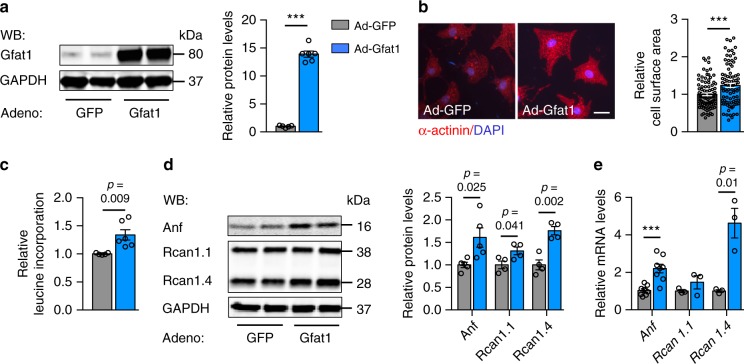
Overexpression of Gfat1 induces hypertrophic growth in cardiomyocytes. **a** NRVMs were infected with adenovirus expressing either control GFP (Ad-GFP) or Gfat1 (Ad-Gfat1). Gfat1 protein level was examined by immunoblotting and quantified. GAPDH was used as a loading control. *N* = 5 for Ad-GFP; *n* = 6 for Ad-Gfat1. **b** Representative images of NRVMs stained for α-actinin (red) and the nucleus (DAPI, blue) are shown. Scale: 20 μm. Bar graph depicts relative fold changes in cardiomyocyte surface area normalized to the cells infected by Ad-GFP. *N* = 91 cells for Ad-GFP; *n* = 86 cells for Ad-Gfat1. At least three independent experiments were conducted with two to three samples/group/experiment. **c** Radioactive leucine was included in culture media after Gfat1 overexpression. Leucine incorporation was quantified as indication of protein synthesis. *N* = 5 for Ad-GFP; *n* = 6 for Ad-Gfat1. **d** Overexpression of Gfat1 in NRVMs led to upregulation of hypertrophic marker genes (Anf, Rcan1.4) at the protein levels. *N* = 5 for Anf; *n* = 4 for Rcan1. **e** The hypertrophic marker gene expression was augmented by Gfat1 overexpression at the mRNA levels, as examined by real-time PCR. *N* = 8 for *Anf*; *n* = 3 for *Rcan1*. Data are shown as mean ± SEM. Student’s *t* test (two-tailed) was conducted to calculate significance. ***p < 0.001. Source data are provided as a Source Data file.

We have shown that multiple enzymes of the HBP are significantly increased in hypertrophic cardiomyocytes. We next asked whether Gfat1, the rate-limiting enzyme of this pathway, was sufficient to trigger hypertrophic growth. We transduced NRVMs with adenovirus to overexpress Gfat1. We found that Gfat1 overexpression was sufficient to augment cardiomyocyte size, compared with the GFP control (Fig. 2a, b). In agreement, Gfat1 overexpression induced cardiomyocyte protein synthesis (Fig. 2c). Further, hypertrophic markers Anf and Rcan1.4^32^ were greatly upregulated at both protein (Fig. 2d) and mRNA levels (Fig. 2e) by Gfat1 overexpression in NRVMs.

Glucosamine is an intermediate product of the HBP (Supplementary Fig. 1a). Previous studies have shown that exposure of cells to glucosamine is another way to induce the HBP flux^33^. As an alternative approach to Gfat1 overexpression in driving HBP, we treated NRVMs with glucosamine, which was sufficient to increase cell size (Supplementary Fig. 4). Importantly, administration of GlcNAc, the final product of HBP, also triggered cardiomyocyte growth (Supplementary Fig. 4). Collectively, these results support that HBP induction is sufficient to stimulate cardiomyocyte growth.

### Gfat1 is required for hypertrophic growth

**Fig. 3:**
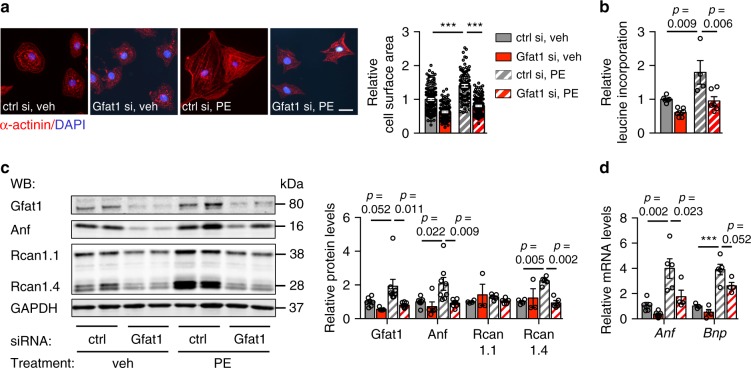
Gfat1 is required for hypertrophic growth in cardiomyocytes. **a** NRVMs were first transfected with either control (ctrl si) or Gfat1 (Gfat1 si) siRNA. PE was then used to trigger cardiomyocyte hypertrophic growth. The cells were harvested for immunofluorescence staining with α-actinin antibody (red) and DAPI (blue). Scale: 20 μm. Quantification showed a significant decrease of cardiomyocyte surface area by Gfat1 silencing. *N* = 160 cells for ctrl si/veh; *n* = 102 cells for ctrl si/PE; *n* = 108 cells for Gfat1 si/veh; *n* = 140 cells for Gfat1 si/PE. At least three independent experiments were conducted with two to three samples/group/experiment. **b** Leucine incorporation assay showed Gfat1 knockdown led to strong suppression of cell growth compared with control siRNA. *N* = 4 for ctrl si/PE group; *n* = 6 for all other groups. **c** Representative immunoblots of Gfat1, Rcan1.1, Rcan1.4, and Anf after PE treatment are shown. Quantification is at the right. *N* = 3–8. **d** Gfat1 silencing in NRVMs inhibited hypertrophic marker expression at the mRNA levels, as examined by real-time PCR analysis. *N* = 3–6. Data are shown as mean ± SEM. Significance was calculated by two-way ANOVA, followed by Tukey’s test. ***p < 0.001. Source data are provided as a Source Data file.

We next asked whether Gfat1 was necessary for hypertrophic growth of cardiomyocytes. We used small interference RNA (siRNA) to knockdown Gfat1 in NRVMs. The cells were then exposed to PE for 48 h. Gfat1 silencing remarkably blunted the hypertrophic effect of PE as shown by significant decreases in cell size (Fig. 3a), protein synthesis (Fig. 3b), and expression of hypertrophic markers (Fig. 3c, d). We confirmed these findings by using another independent siRNA that manifested similar suppression of hypertrophic growth in NRVMs (Supplementary Fig. 5a–d).

We then took another approach to suppress Gfat1. DON (6-diazo-5-oxo-l-norleucine) is a synthetic glutamine analog, which is commonly used as an inhibitor for enzymes utilizing l-glutamine, such as Gfat1. We incubated NRVMs with PE and DON for 48 h to inhibit Gfat1 enzymatic activity (Supplementary Fig. 6a). Indeed, DON treatment led to a significant decrease of cardiomyocyte growth (Supplementary Fig. 6b). Consistently, protein synthesis was suppressed (Supplementary Fig. 6c) and expression of hypertrophic genes was decreased (Supplementary Fig. 6d). Taken together, these data indicate that Gfat1 is required for hypertrophic growth in cardiomyocytes.

### The HBP is induced in the heart by pressure overload

**Fig. 4:**
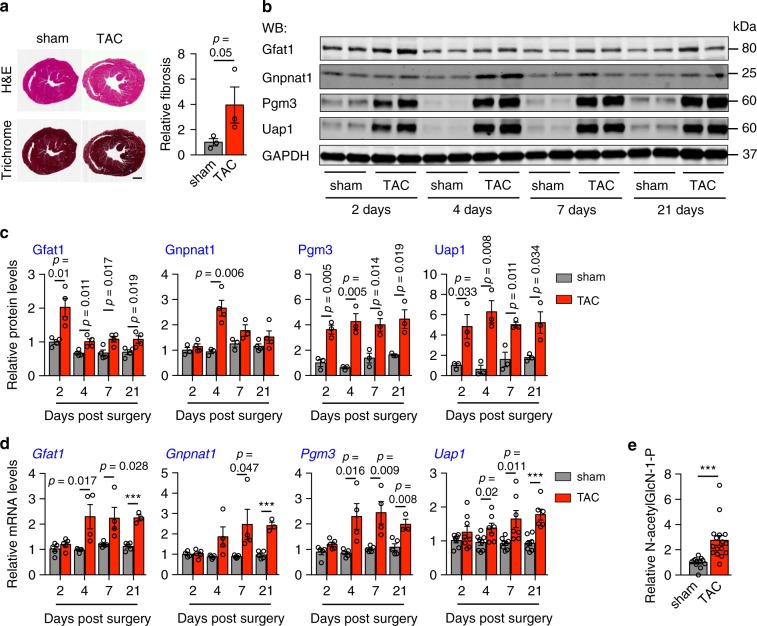
Induction of the HBP by pressure overload in mice. **a** Wild type adult mice were subjected to either sham or thoracic aortic constriction (TAC) surgery. Representative images are shown for heart sections, stained with hematoxylin & eosin and Masson’s trichrome at 21 days after surgery. Scale: 1 mm. Note that fibrosis was significantly elevated (quantified at the right). Mann–Whitney test (one-tailed) was used. *N* = 3. **b** The HBP enzymes (Gfat1, Gnpnat1, Pgm3, and Uap1) in mouse hearts were upregulated at the protein levels in response to pressure overload. GAPDH was used as a loading control. **c** Quantification of **b** showed significant increases at the protein levels of multiple enzymes of the HBP. *N* = 3–4. **d** Cardiac hypertrophic growth by TAC was associated with induction of the HBP enzymes at the mRNA levels as assessed by real-time PCR. *N* = 3–8. **e** N-acetylglucosamine-1-phosphate, an intermediate product of the HBP, was measured by mass spectrometry using cardiac tissues. Note that TAC led to a significant increase in the cardiac level of this metabolite, compared with the sham operation. *N* = 12 for sham; *n* = 16 for TAC. Data are shown as mean ± SEM. Student’s *t* test (two-tailed) was used to evaluate the significance. ***p < 0.001. Source data are provided as a Source Data file.

We have shown induction of the HBP in hypertrophic cardiomyocytes in vitro. Next, we went on to assess whether the HBP was increased in hypertrophic hearts at the in vivo level. We subjected wild type adult mice of 8 weeks old to thoracic aortic constriction (TAC) to induce cardiac hypertrophic growth. Here, we used a 27-G needle to guide narrowing of the aorta, which triggered significant elevation of afterload pressure. In response, the heart was increased in size to accommodate ventricular wall stress. Due to limited regenerative capacity, cardiomyocytes manifested hypertrophic growth to enlarge the heart, which might decompensate and progress into heart failure under persistent stress^10^. TAC surgery is routinely used to model pressure overload and trigger robust, reproducible cardiac hypertrophic growth (Fig. 4a)^34^. We harvested cardiac tissues at different time points after surgery to detect the temporal regulation of the HBP during hypertrophic growth. Multiple enzymes of the HBP were induced at the protein levels in the hypertrophic hearts (Fig. 4b, c). Consistently, the mRNA levels of *Gfat1* and *Pgm3* were significant upregulated as early as 4 days post surgery and remained elevated at 21 days (Fig. 4d). Moreover, we found that cardiac level of N-acetylglucosamine-1-phosphate, an intermediate product of the HBP, was markedly increased (Fig. 4e). Collectively, these data demonstrate that HBP activation in the heart is correlated with pressure overload-induced cardiac hypertrophy in vivo.

### Cardiac-specific overexpression of Gfat1 leads to more profound hypertrophy in response to pressure overload

**Fig. 5:**
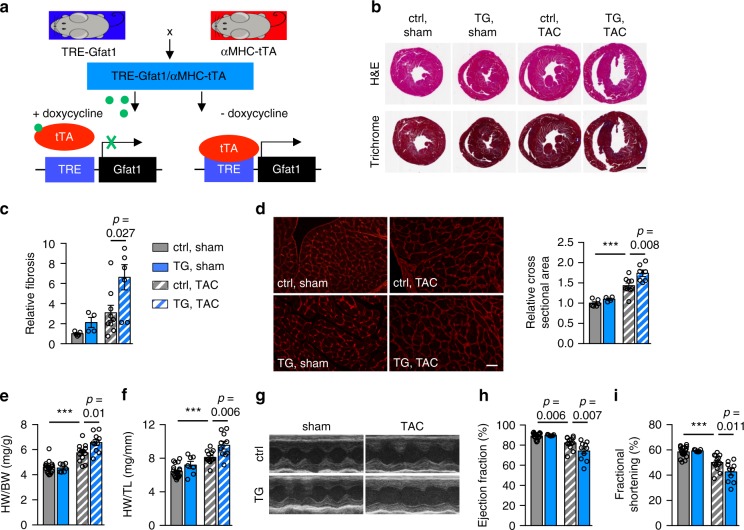
Cardiac-specific overexpression of Gfat1 leads to a more profound hypertrophic response by pressure overload. **a** Schematic representation of TRE-Gfat1 and αMHC-tTA models for generation of the cardiac-specific Gfat1 transgenic mouse model (TG). Control (either TRE-Gfat1 only or αMHC-tTA only) and TG (TRE-Gfat1;αMHC-tTA double transgenic) mice were maintained on doxycycline water to suppress the transgene Gfat1 expression. These mice were switched to regular water for 2 weeks to induce Gfat1 expression and then subjected to surgery. **b** Representative images of mouse heart sections stained with either hematoxylin & eosin or Masson’s trichrome at 3 weeks post sham or TAC. Scale: 1 mm. **c** Quantification showed a significant increase of fibrosis in the Gfat1 TG heart after TAC. *N* = 5 for ctrl/sham; *n* = 4 for TG/sham; *n* = 9 for ctrl/TAC; *n* = 7 for TG/TAC. **d** Wheat germ agglutinin (WGA) staining of cardiac sections (left) showed a significant increase in cardiomyocyte size (quantified at right) in the TG mice after TAC. Scale: 20 μm. *N* = 6 for ctrl/sham; *n* = 4 for TG/sham; *n* = 9 for ctrl/TAC; *n* = 7 for TG/TAC. **e** Measurements of heart weight/body weight ratio (HW/BW) indicate significant cardiac hypertrophic growth in the TG mice. **f** Heart weight/tibia length ratio (HW/TL) suggests that Gfat1 overexpression in the heart led to more profound cardiac hypertrophy by pressure overload. **g** Representative images of M-mode echocardiography. **h** Gfat1 overexpression exacerbated cardiac response by pressure overload as shown by a significant decrease in ejection fraction. **i** Fractional shortening measurement indicates that Gfat1 overexpression led to cardiac dysfunction after TAC in TG mice compared to control animals. *N* = 19 for ctrl/sham; *n* = 7 for TG/sham; *n* = 13 for ctrl/TAC; *n* = 10 for TG/TAC (**e**–**i**). Data are shown as mean ± SEM. Significance was calculated by two-way ANOVA, followed by Tukey’s test. ***p < 0.001. Source data are provided as a Source Data file.

Cardiac hypertrophic growth by pressure overload is associated with profound metabolic remodeling^16^. Previous studies have shown strong elevation of glucose utilization in hypertrophic hearts^22^. The increased use of HBP as a route of glucose metabolism has been noticed^23,24^. However, direct in vivo evidence is missing regarding whether the HBP augmentation is causative or associative. To address this question, we took an inducible approach to upregulate the HBP in a cardiomyocyte-specific manner. We generated a transgenic mouse model with Gfat1 under the control of seven tetracycline responsive elements (TRE-Gfat1) (Fig. 5a). We crossed it to the cardiomyocyte-specific αMHC-tTA transgenic mouse model. In the double transgenic mouse, the transcriptional factor tTA was inhibited by doxycycline and the transgene Gfat1 was not induced. Upon removal of doxycycline from drinking water, tTA was activated and Gfat1 was induced only in cardiomyocytes. We have previously applied this inducible approach in the heart^9,30^, the liver^35^, and the adipose tissue^36^, which represents a tight, efficient, and reproducible means to genetically manipulate gene expression. We supplemented doxycycline (0.1 mg/L) in drinking water during breeding, pregnancy, and weaning. This dose of doxycycline does not affect water consumption or food intake.

We removed doxycycline from drinking water to induce Gfat1 expression in adult mice for 2 weeks. We found that Gfat1 was significantly elevated in the transgenic heart at the protein level (Supplementary Fig. 7a). The Gfat1 transgenic (TG) mice did not show appreciable changes in cardiac histology and no difference in fibrosis was noticed (Supplementary Fig. 7b). In addition, cardiomyocyte size was not altered in transgenic hearts compared to controls (Supplementary Fig. 7c). Heart mass was similar (Supplementary Fig. 7d). Importantly, echocardiography showed indistinguishable cardiac function as revealed by percentage of fraction shortening in TG versus control mice (Supplementary Fig. 7e). Further, transcriptional profiles of multiple genes involved in cardiac hypertrophy, the unfolded protein response, and the HBP did not differ (Supplementary Fig. 7f). We therefore conclude that cardiac-specific Gfat1 overexpression does not affect cardiac function at the baseline.

We next asked whether Gfat1 overexpression might affect cardiac hypertrophic growth and heart failure in response to pressure overload. We used control and Gfat1 TG mice for sham or TAC after Gfat1 expression was turned on for 2 weeks. We first measured the peak aortic velocity across the constriction sites of control and TG mice (Supplementary Fig. 8a). We then calculated pressure gradient, which was approximately 40 mmHg and within the range of previous reports^37,38^. No significant difference was identified between control and TG mice (Supplementary Fig. 8b), suggesting the animals were banded to a similar degree. At the histological level, Gfat1 TG mice manifested increases in heart size (Fig. 5b) and fibrosis (Fig. 5b, c). Wheat germ agglutinin (WGA) staining showed that TG cardiomyocytes were larger than controls following TAC (Fig. 5d). Consistently, TG hearts were bigger as revealed by an increase in heart weight normalized to body weight (HW/BW) or tibia length (HW/TL) (Fig. 5e, f). Further, a significant increase of left ventricular (LV) mass measured by echocardiography was observed in TG mice without changes in heart rate (Supplementary Fig. 9a, b). Gfat1 TG mice also showed a trend of thicker LV internal diameter (LVID) in systole as compared with those in the control group (Supplementary Fig. 9c). No significant differences in interventricular septum (IVS) and LV posterior wall (LVPW) thickness were found, although a trend to increase was noticed (Supplementary Fig. 9d, e). Consistently, LV volume showed a trend of increase in the TG hearts (Supplementary Fig. 9f). At the functional level, representative M-mode echocardiographic images suggested a larger chamber size and defects in contraction in the Gfat1 TG mice after TAC (Fig. 5g). Both ejection fraction and fractional shortening were depressed in TAC-operated Gfat1 TG mice, compared with control animals (Fig. 5h, i). Taken together, these findings suggest that chronic overexpression of Gfat1 and persistent induction of the HBP exacerbate pathological cardiac remodeling and impair cardiac function in response to pressure overload.

### Cardiomyocyte-specific knockout of Gfat1 attenuates pathological remodeling and cardiac dysfunction by pressure overload

We next asked whether cardiac Gfat1 was required for pathological remodeling and heart failure development in response to pressure overload. To answer this question, we generated cardiac-specific conditional knockout animal model for Gfat1. We obtained mice with floxed *Gfat1* alleles (Gfat1^fl/fl^) from the European Mouse Mutant Achieve (EMMA) and crossed them to the cardiac-specific αMHC-Cre transgenic mouse. Out of 98 pups, we were unable to identify viable mice with the Gfat1^fl/fl^;αMHC-Cre genotype, suggesting cardiomyocyte-specific deletion of Gfat1 is embryonically lethal. These data highlight the importance of Gfat1 during cardiac development.

**Fig. 6:**
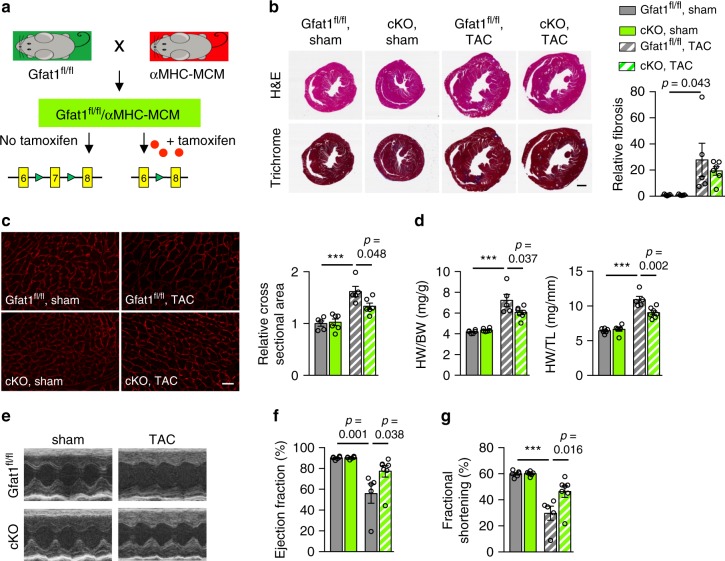
Cardiac-specific Gfat1 deficiency attenuates pathological remodeling and improves cardiac dysfunction in response to pressure overload. **a** Schematic representation of Gfat1^fl/fl^ and α-MHC-MCM models for generation of cardiac-specific inducible Gfat1 conditional knockout mouse model. Both control (Gfat1^fl/fl^ only) and cKO (Gfat1^fl/fl^;αMHC-MCM) animals were injected with tamoxifen for 5 consecutive days, followed by sham or TAC surgery. **b** Representative images of mouse heart sections stained with either hematoxylin & eosin or Masson’s trichrome at 3 weeks after surgery. Scale: 1 mm. Relative fibrosis was quantified at the right. *N* = 6 for cKO/TAC; *n* = 5 for all other groups. **c** Cardiomyocyte WGA staining was conducted using cardiac tissue sections (left). Scale: 20 μm. Bar graph depicts quantification of cross-sectional area normalized to the ctrl/sham group (right). *N* = 5 for Gfat1^fl/fl^; *n* = 6 for cKO. **d** Ratios of heart weight/body weight (HW/BW) and heart weight/tibia length (HW/TL) indicate that Gfat1 deletion suppressed cardiac hypertrophic growth. *N* = 6 for Gfat1^fl/fl^/sham; *n* = 6 for cKO/sham; *n* = 5 for Gfat1^fl/fl^/TAC; *n* = 7 for cKO/TAC. **e** Representative images of M-mode echocardiography. **f** Quantification of ejection fraction showed an improvement in cardiac systolic function in the cKO mice after TAC. *N* = 6 for Gfat1^fl/fl^/sham; *n* = 6 for cKO/sham; *n* = 5 for Gfat1^fl/fl^/TAC; *n* = 7 for cKO/TAC. **g** Fractional shortening measurement indicates that Gfat1 deletion in the heart improved cardiac response by pressure overload. *N* = 6 for Gfat1^fl/fl^/sham; *n* = 6 for cKO/sham; *n* = 5 for Gfat1^fl/fl^/TAC; *n* = 7 for cKO/TAC. Data are shown as mean ± SEM. Significance was calculated by two-way ANOVA, followed by Tukey’s test. ***p < 0.001. Source data are provided as a Source Data file.

We next bred the Gfat1^fl/fl^ mice into the αMHC-MCM background. Under the basal condition, Cre was sequestered in cytoplasma and no excision took place at the *Gfat1* genomic loci. We injected tamoxifen for 5 consecutive days into adult animals to induce nuclear Cre translocation, triggering deletion of *Gfat1* only in cardiomyocytes (Fig. 6a). We verified that tamoxifen treatment led to approximately 90% of *Gfat1* deletion at the DNA level in isolated cardiomyocytes (Supplementary Fig. 10a, b) and at the protein level by approximately 50% (Supplementary Fig. 10c) in the heart. The partial reduction of Gfat1 in cardiac tissue is probably due to expression of Gfat1 in non-cardiomyocytes in the heart. At baseline, cardiac deficiency of Gfat1 (cKO) did not affect the heart at the histological level (Supplementary Fig. 11a). No significant changes in fibrosis were found (Supplementary Fig. 11a). Cardiomyocyte cross-sectional area did not show a difference between control and cKO hearts (Supplementary Fig. 11b). The heart mass was similar (Supplementary Fig. 11c) and cardiac function was maintained (Supplementary Fig. 11d). Moreover, the transcriptional levels of genes related to cardiac hypertrophy, the unfolded protein response, and the HBP were not altered (Supplementary Fig. 11e). The decrease of *Gfat1* mRNA expression in the cKO heart (Supplementary Fig. 11e) is consistent with approximately 50% reduction of the Gfat1 protein level (Supplementary Fig. 10c). Collectively, cardiac-specific deletion of Gfat1 in adult mice does not affect cardiac function and performance at baseline.

We next subjected Gfat1 cKO and control mice to sham or TAC surgery to induce cardiac hypertrophy and heart failure (Supplementary Fig. 12a). Here, we conducted the TAC surgery using a 28-gauge needle to guide the ligation. Previous studies have shown that this severe constriction may trigger more robust pathological remodeling and acceleration of heart failure development within weeks^34^. Examinations were done at 3 weeks following surgery. Histological analysis showed less enlarged hearts in the cKO mice (Fig. 6b). Fibrosis showed a trend of decrease in the cKO hearts compared to controls in response to pressure overload (Fig. 6b). Cardiomyocytes were markedly smaller in cKO mice after TAC (Fig. 6c). Further, the elevation of HW/BW and HW/TL by TAC was significantly decreased in cKO mice than those in the control group (Fig. 6d).

Echocardiographic analyses further supported diminished pathological remodeling by Gfat1 deficiency. Although heart rate was not affected, there was a smaller increase in LV mass of cKO group after TAC in comparison to control mice (Supplementary Fig. 12b, c). Cardiac systolic performance of cKO mice as shown by LVID was remarkably improved compared to controls (Supplementary Fig. 12d). No noticeable changes in IVS and LVPW thickness were detected (Supplementary Fig. 12e, f). Consistent with the decrease of cardiac hypertrophy, cKO mice showed a trend of normalized chamber size and LV volume (Supplementary Fig. 12g). Importantly, cardiac function was preserved after TAC by Gfat1 knockout as revealed by increases in ejection fraction and fractional shortening (Fig. 6e–g). At long-term, Gfat1 knockout mice showed a trend of enhanced survival after TAC. Collectively, cardiac-targeted Gfat1 deletion limits pathological hypertrophy and improves cardiac function in response to pressure overload.

### Gfat1 regulates mTOR signaling

We next sought to delineate the underlying molecular mechanism of Gfat1-induced cardiac hypertrophic growth under pressure overload. mTOR is a serine/threonine kinase, belonging to the PI3K kinase family. mTOR forms two distinct kinase complexes, mTORC1 and mTORC2, which are signaling nexuses coupling hormone actions, intracellular pathways, and cell growth^39^. Extensive evidence has established that mTOR signaling plays a crucial role in cardiac hypertrophy^27,40,41^. We wondered whether the HBP might potentiate hypertrophic growth via stimulation of mTOR in the heart.

**Fig. 7:**
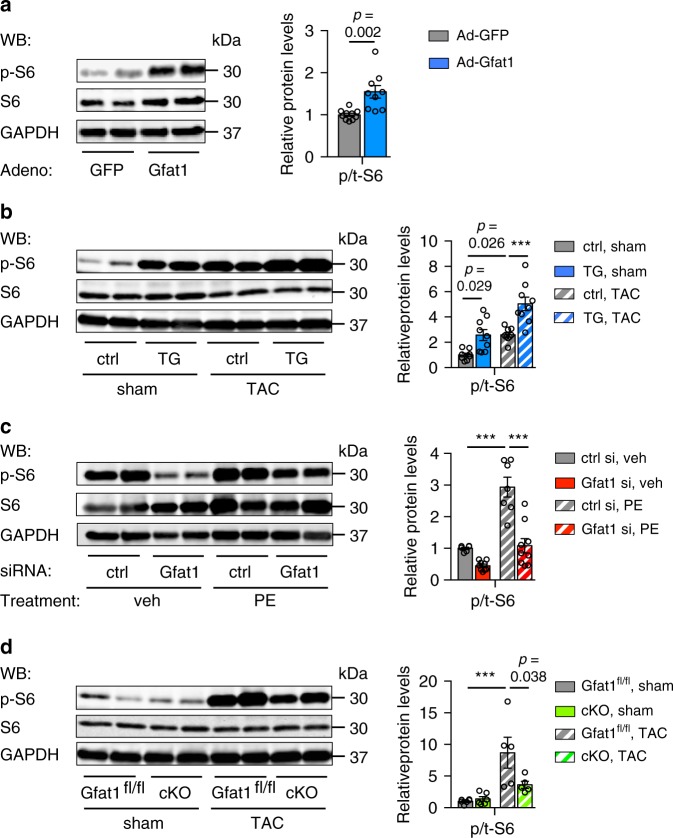
The HBP stimulates mTOR signaling. **a** Gfat1 overexpression in NRVMs was sufficient to activate mTOR signaling as shown by an increase in S6 phosphorylation, a downstream event of the mTOR pathway. *N* = 10 for Ad-GFP; *n* = 9 for Ad-Gfat1. Student’s *t* test (two-tailed) was conducted. **b** Gfat1 overexpression in the heart led to activation of the mTOR pathway. Gfat1 expression was turned on in the double transgenic mice for 2 weeks. After TAC, the heart was harvested to assess mTOR signaling. Pressure overload caused upregulation of S6 phosphorylation that was further potentiated by Gfat1 overexpression. *N* = 8 for ctrl/sham; *n* = 9 for other groups. **c** Gfat1 silencing in NRVMs diminished the mTOR pathway after PE treatment. *N* = 6 for ctrl si/veh; *n* = 9 for Gfat1 si/veh; *n* = 7 for ctrl si/PE; *n* = 9 for Gfat1 si/PE. **d** Cardiac-specific knockout of Gfat1 led to a decrease in the mTOR signaling, as evidenced by reduction in S6 phosphorylation. *N* = 6 for sham; *n* = 5 for TAC. Data are shown as mean ± SEM. Significance was calculated by two-way ANOVA, followed by Tukey’s test. ***p < 0.001. Source data are provided as a Source Data file.

NRVM treatment with various hypertrophic stimuli led to cell growth and stimulation of the HBP (Fig. 1 and Supplementary Figs. 1 and 2). We found that these changes were accompanied by activation of the mTOR signaling, as shown by increased phosphorylation of mTOR and two downstream effectors of the mTOR pathway, S6 and 4EBP1 (Supplementary Fig. 13a–d). Importantly, we showed that overexpression of Gfat1 was sufficient to drive mTOR activation, at both in vitro (Fig. 7a and Supplementary Fig. 14a) and in vivo levels even without hypertrophy stimulation (Fig. 7b and Supplementary Fig. 14b). After TAC, mTOR signaling was strongly upregulated in control hearts, which was further elevated under Gfat1 overexpression (Fig. 7b). On the other hand, siRNA-mediated silencing of Gfat1 significantly diminished the activation of mTOR (Fig. 7c and Supplementary Fig. 14c), which was confirmed by another independent siRNA oligo (Supplementary Fig. 15). Since cardiomyocyte-restricted knockout of Gfat1 led to a decrease in hypertrophic growth after pressure overload, we wondered whether mTOR signaling was affected. Indeed, cardiac Gfat1 deficiency attenuated the activation of mTOR (Fig. 7d and Supplementary Fig. 14d). Consistently, Akt signaling, an upstream activator of mTOR, was reduced (Supplementary Fig. 14d). Taken together, these findings suggest that Gfat1 and HBP may directly stimulate the mTOR pathway to drive cardiac hypertrophic growth under pressure overload.

### mTOR signaling is required for Gfat1-induced hypertrophic growth

**Fig. 8:**
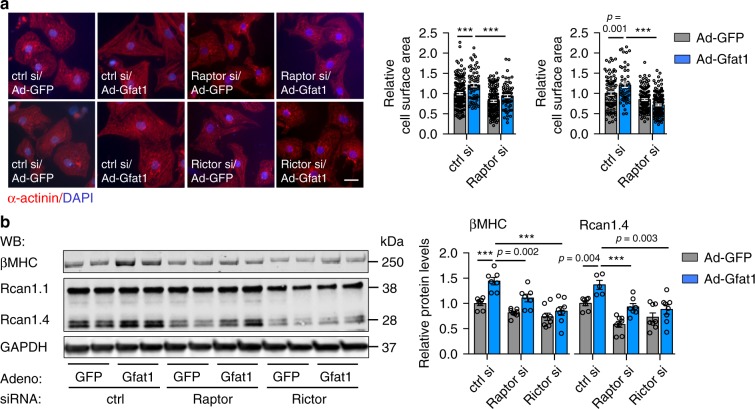
Both mTORC1 and mTORC2 contribute to Gfat1-mediated cardiomyocyte growth. **a** Knockdown of either Raptor or Rictor reduced Gfat1-mediated cardiomyocyte growth. NRVMs were transfected by siRNAs against Raptor or Rictor along with controls. Adenovirus expressing GFP or Gfat1 was then used to infect the cells. After immunofluorescent staining, cardiomyocyte size was quantified (right). Scale: 20 μM. *N* = 55–153/cells for each group. At least three independent experiments were conducted with 2–3 samples/group/experiment. **b** Protein expression of βMHC and Rcan1 was determined by Western blotting. Quantification at the right showed a significant decrease in expression by knockdown of either Raptor or Rictor, indicating both mTORC1 and mTORC2 contribute to Gfat1-mediated cardiomyocyte growth. *N* = 5–8. Data are shown as mean ± SEM. Significance was calculated by two-way ANOVA, followed by Tukey’s test. ***p < 0.001. Source data are provided as a Source Data file.

Since Gfat1 overexpression is sufficient to stimulate mTOR signaling and cardiomyocyte growth, we next asked whether mTOR was required for the pro-hypertrophic effect of Gfat1. mTOR forms two distinct but functionally related complexes, mTORC1 and mTORC2. We first addressed which complex might contribute to the Gfat1 action in cardiomyocytes. We silenced Raptor and Rictor to suppress mTORC1 and mTORC2, respectively (Supplementary Fig. 16a). These cells were then infected by adenovirus expressing either GFP control or Gfat1. Overexpression of Gfat1 led to an increase of cardiomyocyte size, which was significantly suppressed by knockdown of either Raptor or Rictor (Fig. 8a). Consistently, molecular markers of the fetal gene program were reduced by silencing of either Raptor or Rictor at the protein level (Fig. 8b). Moreover, changes in the mRNA level confirmed that both Raptor and Rictor were required for Gfat1-mediated hypertrophic growth (Supplementary Fig. 16b). Taken together, these findings suggest that both mTORC1 and mTORC2 contribute to the action of Gfat1 in cardiomyocyte hypertrophic growth.

**Fig. 9:**
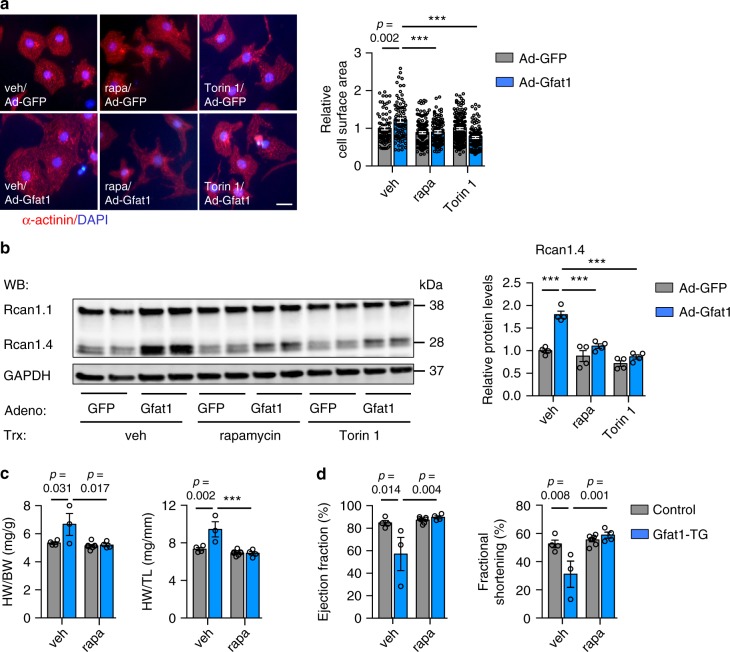
Inhibition of mTOR suppresses Gfat1-induced hypertrophic growth. **a** Pharmacological inhibition of mTOR by rapamycin or Torin 1 prevented cardiomyocyte growth from Gfat1 overexpression. Adenovirus-mediated overexpression of Gfat1 led to an increase in cardiomyocyte size, which was suppressed by either rapamycin (20 nM) or Torin 1 (50 nM) treatment in NRVMs. Scale: 20 μM. *N* = 80 cells for Ad-GFP/veh; *n* = 88 cells for Ad-Gfat1/veh; *n* = 143 cells for Ad-GFP/rapa; *n* = 104 cells for Ad-Gfat1/rapa; *n* = 170 cells for Ad-GFP/Torin 1; *n* = 120 cells for Ad-Gfat1/Torin 1. At least three independent experiments were conducted with two to three samples/group/experiment. **b** Inhibition of mTOR caused a decrease in Rcan1.4 expression in NRVMs. Western blotting was conducted to evaluate the expression Rcan1. In contrast to Rcan1.4, Rcan1.1 expression remained unchanged. *N* = 4. **c** Rapamycin treatment in vivo inhibited the hypertrophic growth by Gfat1 overexpression, as assessed by the ratios of heart weight/body weight (HW/BW) and heart weight/tibia length (HW/TL). Note that all mice underwent TAC surgery. *N* = 4 for control/veh; *n* = 3 for Gfat1-TG/veh; *n* = 7 for control/rapa; *n* = 4 for Gfat1-TG/rapa. **d** Inhibition of mTOR by rapamycin improved cardiac systolic performance in Gfat1 transgenic mice. *N* = 4 for control/veh; *n* = 3 for Gfat1-TG/veh; *n* = 7 for control/rapa; *n* = 4 for Gfat1-TG/rapa. Data are shown as mean ± SEM. Significance was calculated by two-way ANOVA, followed by Tukey’s test. ***p < 0.001. Source data are provided as a Source Data file.

We next used pharmacological inhibitors to suppress mTOR and further delineated the relationship between the mTOR signaling and Gfat1-mediated cardiomyocyte growth. We first overexpressed Gfat1 by adenovirus infection. We then treated NRVMs with rapamycin (20 nM) for 24 h. Rapamycin treatment led to strong suppression of mTOR signaling (Supplementary Fig. 17). Importantly, the Gfat1-mediated increase of cell size was inhibited by rapamycin (Fig. 9a). This was accompanied by a decrease of Rcan1.4 expression (Fig. 9b). Importantly, Torin 1, another inhibitor of mTOR, showed similar inhibitory phenotypes (Fig. 9a, b, and Supplementary Fig. 17).

To further address the role of mTOR in mediating Gfat1 action, we turned to in vivo using the Gfat1 transgenic mouse model. We showed previously that overexpression of Gfat1 led to exacerbation of cardiac response under pressure overload (Fig. 5). We asked whether suppression of mTOR might rescue the cardiomyopathy phenotype. We turned on Gfat1 overexpression by switching to regular drinking water for 2 weeks. We then conducted TAC surgery to induce pressure overload. Rapamycin was administrated at the dose of 2 mg/kg for 3 weeks^42,43^. We found that rapamycin significantly suppressed hypertrophic growth in Gfat1 transgenic mice after TAC (Fig. 9c). Consistently, echocardiography analysis showed a decrease in LV mass (Supplementary Fig. 18a, b). LVID at both systole and diastole was improved by rapamycin treatment (Supplementary Fig. 18c). Although IVS and LVPW did not show significant changes, LV volume was reduced (Supplementary Fig. 18d–f). Importantly, rapamycin treatment improved cardiac function as revealed by increases in both ejection fraction and fractional shortening in the Gfat1 transgenic hearts under pressure overload (Fig. 9d). Taken together, these data suggest that mTOR is required for Gfat1-mediated cardiomyocyte growth, and suppression of mTOR can rescue cardiomyopathy due to persistent elevation of Gfat1 in vivo.

### Gfat1 activates mTOR signaling through O-GlcNAcylation

Our results suggest that Gfat1 is necessary and sufficient for mTOR activation. We next went on to dissect the underlying mechanisms by which Gfat1 stimulated mTOR. Gfat1 is the rate-limiting enzyme of the HBP. The final product of the HBP, UDP-GlcNAc, is involved in multiple biological processes, i.e., proteoglycan and glycolipid synthesis. In addition, UDP-GlcNAc is an obligate substrate for O-GlcNAcylation on serine or threonine sites, a prominent post-translational modification on numerous proteins^2^. Studies have shown that O-GlcNAcylation is sensitive to metabolic fluctuation, which plays critical roles in the regulation of signaling transduction, growth, differentiation, etc.^5,6,8^.

We sought to address whether Gfat1 activated mTOR via upregulation of O-GlcNAcylation. We first examined whether overexpression of Gfat1 in NRVMs might drive the increase of protein O-GlcNAcylation. We infected NRVMs with adenovirus expressing GFP or Gfat1. Immunoblotting showed that Gfat1 overexpression increased O-GlcNAcylation (Supplementary Fig. 19a). On the other hand, PE treatment led to an increase in O-GlcNAcylation, and siRNA-mediated silencing of Gfat1 significantly decreased this protein modification (Supplementary Fig. 19b). At the in vivo level, overexpression of Gfat1 in the heart caused a significant increase of O-GlcNAcylation on cardiac proteins after TAC (Supplementary Fig. 19c). In contrast, conditional knockout of Gfat1 from cardiomyocytes decreased O-GlcNAcylation in the heart after pressure overload (Supplementary Fig. 19d). Collectively, these results indicate that Gfat1 expression is correlated with the level of O-GlcNAcylation.

**Fig. 10:**
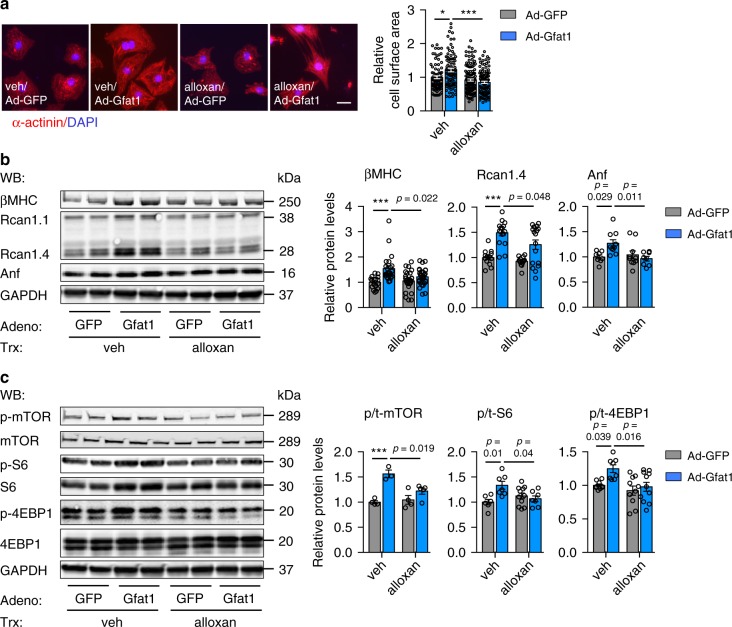
Alloxan inhibits Gfat1-induced hypertrophic growth. **a** Inhibition of O-GlcNAcylation with alloxan led to a decrease in cardiomyocyte growth from Gfat1 overexpression. Adenovirus-mediated overexpression of Gfat1 increased cardiomyocyte size, which was reduced by alloxan treatment in NRVMs. Scale: 20 μM. *N* = 80 cells for Ad-GFP/veh; *n* = 88 cells for Ad-Gfat1/veh; *n* = 135 cells for Ad-GFP/alloxan; *n* = 118 cells for Ad-Gfat1/alloxan. At least three independent experiments were conducted with two to three samples/group/experiment. **b** Suppression of O-GlcNAcylation caused a decrease in the expression of molecular markers of cardiomyocyte hypertrophy in NRVMs. Western blotting was conducted. In contrast to Rcan1.4, Rcan1.1 expression remained unaltered. *N* = 8–29. **c** The mTOR signaling was significantly inhibited by alloxan treatment. *N* = 3–11. Data are shown as mean ± SEM. Significance was calculated by two-way ANOVA, followed by Tukey’s test. *p = 0.003; ***p < 0.001. Source data are provided as a Source Data file.

To further examine the role of O-GlcNAcylation in Gfat1-induced mTOR activation and hypertrophic growth, we used alloxan, a specific inhibitor of O-GlcNAc transferase (OGT). Alloxan treatment led to a significant decrease in protein O-GlcNAcylation (Supplementary Fig. 20a). We found that alloxan diminished Gfat1-induced cardiomyocyte growth (Fig. 10a), along with a decrease in molecular markers of hypertrophy at both protein (Fig. 10b) and mRNA (Supplementary Fig. 20b) levels. Importantly, mTOR activated by Gfat1 overexpression was significantly decreased by alloxan treatment (Fig. 10c). Consistent with the important role of O-GlcNAcylation in mediating Gfat1-induced hypertrophic growth, OGT knockdown led to decreases in O-GlcNAcylation (Supplementary Fig. 21a) and cardiomyocyte growth (Supplementary Fig. 21b). These results together indicate that O-GlcNAcylation is required for Gfat1-induced cardiomyocyte growth and mTOR activation.

Since suppression of Gfat1 decreased cardiomyocyte growth, we next asked whether supplementation of the product of Gfat1 might rescue this phenotype. Before Gfat1 silencing, we treated the NRVMs with glucosamine, the immediate product of Gfat1. We found that cardiomyocyte size was reduced by Gfat1 knockdown, which was significantly rescued by supplementation of glucosamine (Supplementary Fig. 22a). Consistent with the role of Gfat1 in cardiomyocyte growth, inclusion of GlcNAc in culture medium showed a similar rescue effect (Supplementary Fig. 22b). Furthermore, using TMG (Thiamet-G) to boost O-GlcNAcylation in NRVMs led to a significant increase in cardiomyocyte size (Supplementary Fig. 22c). Importantly, these treatments were associated with elevation of O-GlcNAcylation (Supplementary Fig. 22d). Taken together, these findings suggest that Gfat1, the HBP, and consequent O-GlcNAcylation play critical roles in governing cardiomyocyte growth.

## Discussion

Hypertension is one of the most important risk factors of heart failure. In response to high blood pressure, cardiac ventricular wall stress is augmented and the heart manifests hypertrophic growth to accommodate the increased demand. This once adaptive response may decompensate and succumb to heart failure under persistent stress. Pathological hypertrophic growth involves remodeling at various levels, including metabolism, structure, electrophysiology, etc. Ample evidence suggests that metabolic alteration is one of the most prominent changes and precedes most other remodeling events. This metabolic remodeling is a continuum, exemplified by an early increase in glucose utilization and a decrease in fatty acid catabolism. At the late stage of hypertensive cardiomyopathy, reduction of both glucose and fatty acid metabolism has been identified^44^. Here, we show that the HBP, one of the glucose metabolic pathways, is strongly and acutely induced in the heart by pressure overload. More importantly, this elevation persists throughout the remodeling phase. These findings suggest that chronic HBP induction may be an underlying mechanism of pathological cardiac remodeling. Indeed, transgenic overexpression of Gfat1, the rate-limiting enzyme of HBP, in the heart exacerbates cardiac dysfunction and heart failure by pressure overload. In contrast, cardiomyocyte-specific deletion of Gfat1 protects the heart from pressure overload-induced cardiac adversities. At the mechanistic level, we show that elevation of the HBP directly stimulates mTOR signaling whereas silencing of Gfat1 significantly suppresses mTOR and cell growth. Taken together, our findings suggest that chronic, persistent HBP induction in the heart by pressure overload may directly drive pathological cardiac remodeling by activating the mTOR pathway.

Gfat1 generates UDP-GlcNAc for O-GlcNAcylation, a prominent post-translational protein modification. Acute induction of the HBP and O-GlcNAcylation by stress has been proposed as an adaptive response^45–47^, while at long-term it may become maladaptive^24^. Indeed, we previously found that acute upregulation of the HBP and O-GlcNAcylation couples with the unfolded protein response to protect cardiomyocyte from ischemia/reperfusion injury^9^. Moreover, the beneficial effect of this activation has been revealed by its ability to restore mitochondrial membrane potential^47,48^ and reduce mitochondrial oxidative stress^46,49^. On the other hand, diabetes is exemplified by chronic over-nutrition and persistent metabolic challenge. Diabetes-related cardiomyopathy has been partly attributed to long-term induction of the HBP and O-GlcNAcylation in the heart^6,7^. Indeed, previous studies have shown that the elevation of free fatty acids may be able to stimulate the HBP in muscle^50^. On the other hand, activation of the HBP enhances fatty acid metabolism^51^, which may create a vicious cycle of long-term metabolic imbalance. However, the role of HBP in cardiac hypertrophic growth and pathological remodeling under pressure overload is poorly defined.

Early studies have shown an increase of UDP-GlcNAc in the heart by pressure overload; however, the role for this alteration remains elusive^52^. Using both in vitro cell culture and in vivo animal models, we show that the HBP enzymes are significantly elevated in cardiomyocytes during hypertrophic growth. In line with these findings, we found that an intermediate product of the HBP (N-acetylglucosamine-1-phosphate) is upregulated in the heart by pressure overload that remains elevated at 3 weeks post TAC. The genetically engineered mouse models of gain- and loss-of-function allowed us to further define the role of the HBP. Although overexpression of Gfat1 (via adenovirus) in primary cardiomyocyte culture is sufficient to promote hypertrophic growth, the TG mice do not show significant differences in cell size, heart morphology, and cardiac function at baseline. Notably, Gfat1 overexpression exacerbates pathological hypertrophic growth and cardiac dysfunction after pressure overload. Our findings are consistent with the high O-GlcNAc level observed in hypertensive rats and aortic stenosis patients^24^. HBP and O-GlcNAcylation induction may therefore be a maladaptive response to trigger pathological cardiac remodeling.

On the contrary, Gfat1 deficiency in cardiomyocytes blunts hypertrophic growth as revealed by both genetic and pharmaceutical approaches. It should be emphasized that no Gfat1 cardiac knockout mice from embryonic stage survived until weaning. These findings highly suggest that Gfat1 is essential for cardiogenesis during development. Therefore, we turned to a conditional approach by breeding Gfat1^fl/fl^ mice to αMHC-MCM animals to obtain the Gfat1^fl/fl^;αMHC-MCM genotype (inducible, conditional, cardiac-specific Gfat1 knockout). At baseline, Gfat1 cKO mice were indistinguishable from controls in heart morphology, cardiac function, and gene-expression profiles. Importantly, Gfat1 deficiency confers cardioprotection against pathological remodeling and cardiac dysfunction by pressure overload.

The HBP/Gfat1/O-GlcNAcylation axis has emerged as a crucial nutrient and stress-sensing pathway^2^. We have shown that XBP1s is an upstream transcriptional factor of multiple HBP enzymes^9^. Cardiac ischemia/reperfusion induces XBP1s expression and enhances the HBP flux, resulting in elevation of UDP-GlcNAc and O-GlcNAcylation. This acute response confers strong cardioprotection against reperfusion injury^9^. In addition, in response to amino acid deprivation, the GCN2-eIF2α pathway is activated that stimulates ATF4 to drive Gfat1 expression as well as an increase in O-GlcNAcylation^42^. This transient induction of Gfat1 and the HBP may provide survival advantage by enhancing glucose assimilation and driving restoration of metabolic homeostasis. Under conditions of chronic stress, however, this prolonged response may lead to adverse consequences. Recently, AMPK was shown to phosphorylate Gfat1 and suppress its enzymatic activity under pressure overload^25^. Indeed, glucose infusion in the heart leads to stimulation of the HBP, which is associated with pathological remodeling^27^. Here, we show that chronic overexpression of Gfat1 in the heart exacerbates cardiac hypertrophic response and potentiates heart failure development. Although our studies highlight the importance of cardiomyocyte HBP, the increase of the HBP and O-GlcNAcylation may also be contributed from other cell types in the heart. Studies have shown that hypertension is associated with an increase of leukocyte counts^53^, which manifests activation of the HBP and O-GlcNAcylation under metabolic stress^54^. Future work is warranted to dissect the contribution of leukocytes in hypertensive heart disease. Taken together, these findings indicate that persistent elevation of HBP in the heart is maladaptive that contributes to pathological cardiac remodeling in response to pressure overload.

The mTOR signaling is well known to play important roles in hypertrophic growth in both physiological and pathological hypertrophy^40^. Under cardiac stress, carbohydrate utilization is enhanced while fatty acid metabolism is inhibited. Importantly, dysregulation of glucose metabolism activates mTOR, triggers ER stress, and leads to cardiac dysfunction under high workload^27^. On the other hand, the altered glucose utilization is concurrent with the elevated level of UDP-GlcNAc in response to pressure overload^52^. Our findings here suggest that Gfat1 upregulation directly stimulates the mTOR pathway, which therefore provides an underlying mechanistic link between glucose metabolism, mTOR activation, and pathological cardiac remodeling under chronic pressure overload. In conclusion, persistent induction of Gfat1 in the heart may directly activate mTOR signaling, induce pathological cardiac hypertrophy, and exacerbate cardiomyopathy under hemodynamic stress.

## Methods

### Animals

All mice are on the C57BL/6 background. Mice were maintained on a 12 h light/dark cycle from 6 A.M. to 6 P.M. with unrestricted access to food (Teklad, #2916) and water. All animal procedures have been approved by the Institutional Animal Care and Use Committee of University of Texas Southwestern Medical Center (UTSW).

### Generation of cardiac-specific Gfat1 overexpression mouse model

*Gfat1* (NM_013528) was amplified from a mouse heart cDNA library and cloned into the pTRE (tetracycline responsive element) vector (Clontech) with a rabbit β-globin 3′UTR. After sequencing confirmation at both ends, the TRE-Gfat1 transgene was liberated by enzymatic digestion using Nae I, Ahd I, and Not I. The purified TRE-Gfat1 fragment was subjected to pronuclear injection by the Transgenic Technology Center at UTSW. The founder lines were crossed with the αMHC-tTA mouse model^55^. Double transgenic mice were maintained on doxycycline water (0.1 mg/L) to suppress transgene Gfat1 expression (“tet-off”) during breeding, pregnancy, and postnatal growth. We validate that this dose of doxycycline does not affect food consumption and water intake. After weaning, replacement of doxycycline water with regular drinking water for 2–4 weeks led to the activation of transcription factor tTA and consequent induction of Gfat1 expression, exclusively in cardiomyocytes in the heart. This inducible system has been used for various gene inductions in the heart^9,30^, the liver^35^, the hypothalamus^56^, and the adipose tissues^36^, which represents a tight, adjustable, and reproducible approach for in vivo gene overexpression.

### Generation of cardiac-specific Gfat1 conditional knockout mouse model

The Gfat1^tm1a(EUCOMM)Wtsi^ strain was obtained from the Infrafrontier consortium (#EPD0069_2_H11). Gfat1^tm1c(EUCOMM)Wtsi^ and Gfat1^tm1d(EUCOMM)Wtsi^ strains were generated at UTSW. Gfat1^tm1c^ strain (hereinafter referred to as Gfat1^fl/fl^) was produced by crossing Gfat1^tm1a^ with the Flippase transgenic mice to remove the neomycin and LacZ cassettes. The Gfat1^fl/fl^ mouse was then crossed with the cardiomyocyte-specific αMHC-Cre or αMHC-MCM mouse model to generate cardiac-specific conditional knockout of *Gfat1* by excising exon 7. In the Gfat1^fl/fl^;αMHC-Cre mice, *Gfat1* deletion happened during embryonic development and no viable progenies were found.

In the adult Gfat1^fl/fl^;αMHC-MCM mice, DNA recombination of the floxed *Gfat1* alleles was induced by tamoxifen (Sigma, #T5648) injection (I.P.) for 5 consecutive days. To check the efficiency of cardiac specific deletion, genomic DNA isolated from mouse adult cardiomyocytes after 5 consecutive tamoxifen injections (20 mg/kg body weight/day) was used for polymerase chain rection (PCR) analysis. Cardiac function was monitored periodically (2 weeks, 4 weeks) after tamoxifen induction. The animals that recovered from transient cardiac dysfunction were used for sham or TAC surgery. All primers used for genotyping are provided in Supplementary Table 1.

### Cardiomyocyte isolation and treatment

NRVMs were isolated from ventricles of 1–2 days old Sprague–Dawley rats (Charles River Laboratories). Isolation was done in accordance with the instruction of the cardiomyocyte isolation kit (Cellutron, #NC-6031). Cardiomyocytes were plated in plating medium consisting of DMEM/M199 (3:1), 5% fetal bovine serum (FBS), 10% horse serum, 1% penicillin/streptomycin, and 100 µM bromodeoxyuridine. After 24 h, cells were washed and cultured in reduced-serum medium (DMEM/M199, 1% FBS, 1% penicillin/streptomycin, and 100 µM bromodeoxyuridine). NRVMs were then kept in serum-free medium and subjected to various treatments with hypertrophic stimuli, including PE (50 µM) for 24 or 48 h, endothelin-1 (ET-1, 10 nM) for 24 h, insulin-like growth factor-1 (IGF-1, 10 nM) for 24 h, and angiotensin II (Ang II, 1 µM) for 24 h. Either glucosamine (GlcN, 5 mM) or GlcNAc (5 mM) was utilized to activate hexosamine biosynthesis while 6-diazo-5-oxo-L-norleucine (DON, 20 µM) was used to inhibit Gfat1. Thiamet G (TMG, 10 µM) was used to increase O-GlcNAcylation levels whereas Alloxan (2.5 mM) was used to suppress O-GlcNAcylation. To inhibit mTOR signaling, either Rapamycin (20 nM) or Torin 1 (50 nM) was used.

Adult cardiomyocytes were isolated from hearts of 8–16 weeks old male mice^57^. The isolated cells were plated in plating medium consisting of MEM, 2 mM l-glutamine, 1.26 mM CaCl_2_, 25 mM blebbistain, 1% penicillin/streptomycin, and 10% FBS. After 2 h, cells were washed and cultured in serum-free medium (MEM, 2 mM L-glutamine, 1.26 mM CaCl_2_, 25 mM blebbistatin, 1% penicillin/streptomycin, and 0.1% bovine serum albumin (BSA)). The cells were then treated with PE (50 µM) for 24 h.

### Gfat1 overexpression in NRVMs by adenovirus transduction

Adenovirus expressing Gfat1 was purchased from Vector Biolabs (#ADV-260051). GFP-expressing adenovirus was used as a negative control. NRVMs were infected with adenovirus for 16–24 h. Culture medium was then replenished. PE was included to induce hypertrophy for 24 h.

### Knockdown in NRVMs by siRNA transfection

The siRNA oligos against Gfat1, Raptor, Rictor, and OGT were obtained from Sigma. The MISSION^®^ universal negative control siRNA was used as control. Multiple independent siRNA oligos for Gfat1, Raptor, Rictor, and OGT were chosen to avoid sequence-dependent, non-specific effects. NRVMs were transfected with siRNA for 16–24 h using Lipofectamine RNAiMAX (ThermoFisher, #13778075).

### Leucine incorporation assay

Radioactive L-[3,4,5-^3^H]-leucine (PerkinElmer, #NET460A001MC, 2 µCi/mL) was used to quantify amino acid incorporation as a surrogate measure of protein synthesis. After treatments with hypertrophic stimuli, NRVMs were washed twice with ice-cold phosphate-buffered saline (PBS) and then incubated with ice-cold trichloroacetic acid (LabChem, #LC262302) at 4 °C for 30 min. Ice-cold ethanol (95%) was used to wash the cells twice. Subsequently, NaOH (0.5 N, 1 mL/well of 6-well plates) was added and incubated at 37 °C for 24 h. HCl (0.5 N, 1 mL) was then used to neutralize the pH. Radiolabeled leucine incorporation was detected using a liquid scintillation counter (Beckman, #LS5000TA).

### RNA isolation and real-time PCR

Total RNA was isolated from NRVMs and hearts using the Quick-RNA MicroPrep kit (Zymo Research, #R1055) and the Aurum total RNA fatty and fibrous tissue kit (Bio-Rad, #7326870), respectively. The cDNA synthesis was carried out using the iScript reverse transcription Supermix (Bio-Rad, #1708841). Transcriptional levels of various genes were determined using LightCycler 480 (Roche), Bio-Rad CFX96, or Bio-Rad CFX384 (Maestro 1.1) with the SYBR Green qPCR master mix (Biotool, #B21203). Data were normalized to the internal control 18s rRNA. Relative mRNA levels were quantified using the comparative 2^−ΔΔCt^ method. All real-time PCR primers are provided in Supplementary Table 1.

### Immunoblotting analysis

Total proteins were extracted from NRVMs and hearts using the RIPA lysis and extraction buffer (ThermoFisher, #89900). Protein concentrations were determined using a BCA kit (ThermoFisher, #23225). Equal amount of proteins was loaded on Criterion TGX precast gels of 4-20% (Bio-Rad, #5671095) and transferred onto the nitrocellulose membrane (Bio-Rad, #1704157). After blocking, the membrane was incubated with appropriate antibodies for 16–24 h. Bound primary antibodies were then incubated with fluorescent dye-labeled secondary antibodies and detected by an Odyssey infrared image scanner (Li-Cor). The following antibodies were used: Gfat1 (Santa Cruz Biotechnology, #sc-134894), GalE (Abcam, #ab155997), Gnpnat1 (Sigma, #HPA044647), Pgm3 (Sigma, #WH0005238M1), Uap1 (Sigma, #SAB1406469), GAPDH (Fitzgerald, #10R-G109A), p-Akt (Cell Signaling, #9271), Akt (Cell Signaling, #2920), mTOR (Cell Signaling, #4517), p-mTOR (Cell Signaling, #2974), S6 (Cell Signaling, #2217), p-S6 (Cell Signaling, #5364), 4EBP1 (Cell Signaling, #9644), p-4EBP1 (Cell Signaling, #2855), Anf (Abcam, #ab180649), βMHC (Abcam, #ab124205), RCAN1 (Sigma, #D6694), O-GlcNAc (ThermoFisher, MA1-072), IRDye 800 CW goat anti-rabbit secondary antibody (Li-Cor, #925-32211), and Alexa Fluor 700-conjugated goat anti-mouse secondary antibody (ThermoFisher, #A-21036). The Gfat1 antibody was validated by using Gfat1 transgenic hearts, Gfat1 overexpressing NRVMs, and Gfat1 silencing NRVMs. All primary antibodies were used at 1:1000. All secondary antibodies were used at 1:10,000. Full blots can be found in a Source Data file.

### Immunohistochemistry

Cardiac tissues were fixed in 4% paraformaldehyde (PFA) for 16–24 h and dehydrated in 70% ethanol. The hearts were then embedded in paraffin and sectioned at 5-μm thickness. Hematoxylin & eosin staining and Masson’s trichrome staining were done by the Molecular Pathology Core at UTSW. Fibrosis was quantified using the Image J 1.52P software.

### Immunofluorescence

For WGA staining, deparaffinized heart sections were rehydrated and incubated in blocking buffer (5% normal goat serum, 1% BSA in PBS) for 1 h. Cell membrane was stained with Alexa Fluor 594-conjugated WGA (ThermoFisher, #W11262, 10 μg/mL) for 1 h and washed three times in PBS before imaging.

For α-actinin staining in cell culture, NRVMs were washed twice in PBS and then fixed in 4% PFA at 4 °C for 30 min. Cells were washed another three times in PBS and permeabilized in 0.1% Triton X-100 on ice for 5 min. After three washes in PBS, NRVMs were incubated in blocking buffer (1.5% normal goat serum, 1% BSA in PBS) for 1 h and then in primary anti-α-actinin antibody (Abcam, #ab7732) for another hour. The cells were washed in PBS for three times and incubated with Alexa Fluor 568-conjugated goat anti-mouse secondary antibody (ThermoFisher, #A-11031). Excess antibody was removed by three washes of PBS. All slides were then mounted with ProLong Gold antifade mountant with DAPI (ThermoFisher, #P36935) and imaged with a fluorescent microscope (Leica). Cross-sectional area (cardiac tissues) and cell surface area (NRVMs) were analyzed using the Image J 1.52P software.

### Transverse aortic constriction (TAC) surgery

To induce cardiac hypertrophic growth, constriction at the thoracic aorta to the 27- or 28-gauge needle thickness was performed^28,30^. Compared to a 27-gauge needle, constriction to a 28-gauge needle led to more severe, decompensated hypertrophy and heart failure^34^. Sham animals underwent a similar procedure with the exception of ligature constriction. Age- and weight-matched male mice were subjected to surgery. The surgery was done in a blinded manner. Mice were followed for up to 8 weeks post-operation. Cardiac function was monitored by echocardiography. The heart was then collected, weighed, and used for further analysis. Mouse body weights before surgery and at sacrifice are shown in Supplementary Table 2.

### Administration of rapamycin

Rapamycin (2 mg/kg/day) or vehicle was intraperitoneally injected to sham or TAC-operated mice (including control and Gfat1 transgenic groups). Rapamycin or vehicle treatment was started 3 h before surgery, and then continued daily for 3 weeks. Cardiac function was monitored by echocardiography.

### Echocardiography

Heart function was determined in unconstrained, conscious mice using echocardiography (VisualSonics, #Vevo 2100, MS400C probe)^9^. Images were obtained from the parasternal short axis (M-mode). Doppler imaging was used to measure pressure gradient across the aortic constriction site in a noninvasive manner^28^. Measurements were analyzed using the Vevo 2100 image software.

### Statistical analysis

All data are presented as mean ± SEM (standard error of the mean). Student’s unpaired *t* test (two-tailed) was performed to compare differences between two groups. For comparison of more than two groups, one-way ANOVA was conducted, followed by Tukey’s test. In addition, two-way ANOVA was conducted for multiple group comparison if there are ≥2 independent variables, followed by Tukey’s test. A *p* value of <0.05 was considered statistically significant. Data were calculated with Microsoft Excel 14.7.7. Statistical analysis was performed using Graphpad Prism software 7.01.

### Reporting summary

Further information on research design is available in the Nature Research Reporting Summary linked to this article.

## Supplementary information

Supplementary Information

Reporting Summary

## Data Availability

The source data underlying Figs. 1a, b, 2a–e, 3a–d, 4a, 4c–e, 5c–f, 5h, i, 6a–d, 6f–g, 7a–d, 8a, b, 9a–d, 10a–c and Supplementary Figs. 1b–d, 2a–c, 3a, b, 4, 5a–d, 6a–d, 7a–f, 8b, 9a–f, 10c, 11a–e, 12b–g, 13a–d, 14a–d, 15, 16a, b, 17, 18a–f, 19a, 19c, d, 20a, b, 21a, b, 22a–d are provided as a Source Data file. Unprocessed gel images for Figs. 1a, 2a, 2d, 3a, 4b, 7a–d, 8b, 9b, 10b, c and Supplementary Figs. 5d, 7a, 10b, c, 13a–d, 14a–d, 15, 17, 19a–d, 20a, 21a, 22d are provided as a Source Data file. All the other data supporting the findings of this study are available from the corresponding author in reasonable request.

## Source data

Source data
