# Supplementary material for: Chronic activation of hexosamine biosynthesis in the heart triggers pathological cardiac remodeling
Source: Nat Commun. 2020 Apr 14;11:1771. doi: 10.1038/s41467-020-15640-y (PMC7156663; doi:10.1038/s41467-020-15640-y)
Supplement: Supplementary file 1 — Supplementary Information [file 41467_2020_15640_MOESM1_ESM.pdf]

Supplementary Information

Chronic Activation of Hexosamine Biosynthesis in the Heart Triggers Pathological Cardiac Remodeling

Tran *et al*.

Supplementary Table 1. Primers used in this study.

Gene	Species	Primer	Test
TRE-Gfat1	mouse	TAAGGAGATCCAGCGGTGTC/CAGCTGTCTCGCCTGATTGA	genotyping
Cre		GATTTCGACCAGGTTCGTTC/GCTAACCAGCGTTTTCGTTC	genotyping
MCM		ACCTGCTCCTGGAGATGTTG/CATCACTCGTTGCATCGACC	genotyping
$Gfat1^{fl/fl}$	mouse	GGTGTTAACAGGGAGCCATC/GCTCCCGTTCCAATACTCAA	genotyping
$Gfat1^{fl/fl,}$ post recombination	mouse	AAGGCGCATAACGATACCAC/ACTGATGGCGAGCTCAGACC	genotyping
$Gfat1^{fl/fl,}$ post recombination	mouse	GGTGTTAACAGGGAGCCATC/TCGGAATCTCTGACACGC	genotyping
tTA		TCAGACCGAGATTTCTCCATCCC GAATTCAGGCTCGCCTGCAGTTGG	genotyping
18s	rat	AAACGGCTACCACATCCAAG/CCTCCAATGGATCCTCGTTA	qPCR
Gfat1	rat	AGTTGGCACAAGGCGAGGTA/ACGGCACTTGCATCAGAAGC	qPCR
Gnpnat1	rat	GTGGCTACAGCAACTCTGAT/GGAACCTCCGACACATGTAA	qPCR
Pgm3	rat	GGACTGTCGGTTCTGCTGTT/AGCGTCGCTATCTTGTCTCC	qPCR
Uap1	rat	CACCTGCTGCAGTTCTGGAGT/GCAGCTGGTCTTGATCTCTGG	qPCR
GalE	rat	CCTACGGCAAGTCCAAGTTC/CCGTAGCGTAGTCATCACCA	qPCR
Anf	rat	CTTCTTCCTCTTCCTGGCCT/TTCATCGGTCTGCTCGCTCA	qPCR
Rcan1.1	rat	GACCCGCGCGTGTTC/TGTCATATGTTCTGAAGAGGGAATC	qPCR
Rcan1.4	rat	CCCGTGAAAAAGCAGAATGC TCCTTGTCATATGTTCTGAAGAGGG	qPCR
Bnp	rat	TCCTTAATCTGTCGCCGCTG/AGGCGCTGTCTTGAGACCTA	qPCR
βMHC	rat	CTGAGGAGACACAGCGTTCT/GGGTCAGCTGAGAGATAAGA	qPCR
Sma	rat	AGACCACCGCTCTTGTGTGT/GTCAGGATACCTCGCTTGCT	qPCR
Sma	mouse	AGACCACCGCTCTTGTGTGT/GTCAGGATACCTCGCTTGCT	qPCR
18s	mouse	AGGGTTCGATTCCGGAGAGG/CAACTTTAATATACGCTATTGG	qPCR
Gfat1	mouse	TAAGGAGATCCAGCGGTGTC/CAGCTGTCTCGCCTGATTGA	qPCR
Gnpnat1	mouse	AGAAGTGGACTGGAGTCAGA/GGTCACATCTTCCACAACTG	qPCR
Uap1	mouse	ACCTGCTGCAGTTCTGGAATG/CAGCTGCTCTTGATCTCTGGT	qPCR
Pgm3	mouse	TGAGAGATGCTGCTCCTTCG/CTTCTTCAAGGTCCCGCGTG	qPCR
Anf	mouse	CTTCTTCCTCGTCTTGGCCT/CTGCTTCCTCAGTCTGCTCA	qPCR
Bnp	mouse	CATGGATCTCCTGAAGGTGC/CCTTCAAGAGCTGTCTCTGG	qPCR
αMHC	mouse	CGGAACAAGACAACCTCAAT/TGGCAATGATTTCATCCAGC	qPCR
βMHC	mouse	AAGCAGCAGTTGGATGAGCG/CCTCGATGCGTGCCTGAAGC	qPCR
XBP1s	mouse	GGTCTGCTGAGTCCGCAGCAGG GAAAGGGAGGCTGGTAAGGAAC	qPCR
GRP78	mouse	CCTCTCTGGTGATCAGGATA/CGTGGAGAAGATCTGAGACT	qPCR
Gfat2	mouse	AGCCATCCAGACCTTGCAGA/CACAATGAGCCTTCGGCATC	qPCR
Ogt	mouse	GAGTGAAGGTGATGGCGGAA/ATGGCCTGAATAGGAGCTGG	qPCR
Oga	mouse	AGCGAAGATGGCAGAGGAGT/CCGTGCTCGTAAGGAAGGTA	qPCR
Ire1	mouse	GCGATGGACTGGTGGTAACT/TCTTGGCCTCTGTCTCCTTG	qPCR
GRP94	mouse	GATGGTCTGGCAACATGGAG/CGCCTTGGTGTCTGGTAGAA	qPCR

Supplementary Table 2. Mouse body weights before surgery and at the end-point upon sacrifice.

Control and TG mice for 3-week sham or TAC

		body weight (grams)																		
	pre-surgery	25.7	20.1	21.2	20.9	20.7	20.3	20.3	23.2	23.5	23.9	23.0	23.2	26.6	22.3	20.6	22.9	24.0	22.8	20.7
ctrl, sham	*end-point*	28.1	21.5	21.4	19.9	21.4	20.2	23.1	27.6	25.9	25.0	23.8	25.5	28.8	22.4	22.9	26.8	26.9	23.7	24.5
TG, sham	pre-surgery	20.7	23.1	25.3	24.3	23.7	24.3	22.5												
	end-point	22.4	23.7	29.9	29.1	29.2	31.0	28.1												
	pre-surgery	21.8	21.9	20.1	20.9	23.7	25.5	22.1	22.0	23.3	22.4	21.5	24.9	20.1						
ctrl, TAC	*end-point*	23.4	22.3	21.1	23.6	25.4	26.7	22.5	23.7	25.9	26.3	24.7	28.2	23.5						
	pre-surgery	26.1	21.4	20.6	23.7	21.5	20.7	21.5	19.3	20.7	22.2									
TG, TAC	*end-point*	28.2	24.4	24.2	24.7	22.4	24.8	25.5	22.8	25.5	25.5									

Control and TG mice for 3-week sham or TAC, vehicle or rapamycin treated

		body weight (grams)						
ctrl, sham,	*pre-surgery*	23.9	24.9	24.4				
rapamycin	*end-point*	23.0	24.1	24.1				
ctrl, TAC,	*pre-surgery*	25.6	23.7	23.3	23.3			
veh	*end-point*	24.8	23.4	24.0	23.1			
ctrl, TAC,	*pre-surgery*	23.4	26.7	24.1	23.6	24.3	24.9	22.9
rapamycin	*end-point*	22.6	24.9	22.2	22.2	23.4	22.8	22.9
TG, TAC,	*pre-surgery*	23.4	26.2	22.5				
veh	*end-point*	23.6	27.1	23.2				
TG, TAC,	*pre-surgery*	23.1	25.8	22.2	24.3			
rapamycin	*end-point*	22.6	23.4	21.0	23.0			

Control and cKO mice for 3-week sham or TAC

		body weight (grams)						
	pre-surgery	28.1	29.3	29.8	27.4	28.1	27.4	
ctrl, sham	*end-point*	27.4	26.9	28.9	25.8	29.4	26.4	
	pre-surgery	24.2	26.9	28.8	28.6	29.7	28.9	
cKO, sham	*end-point*	22.4	25.6	27.7	28.7	29.3	27.9	
	pre-surgery	25.9	26.9	28.2	27.5	26.1		
ctrl, TAC	*end-point*	24.8	29.4	24.2	29.5	28.4		
	pre-surgery	29.6	26.5	27.3	24.4	27.2	24.8	24.8
cKO, TAC	*end-point*	28.9	26.5	26.4	23.9	27.9	24.0	26.6

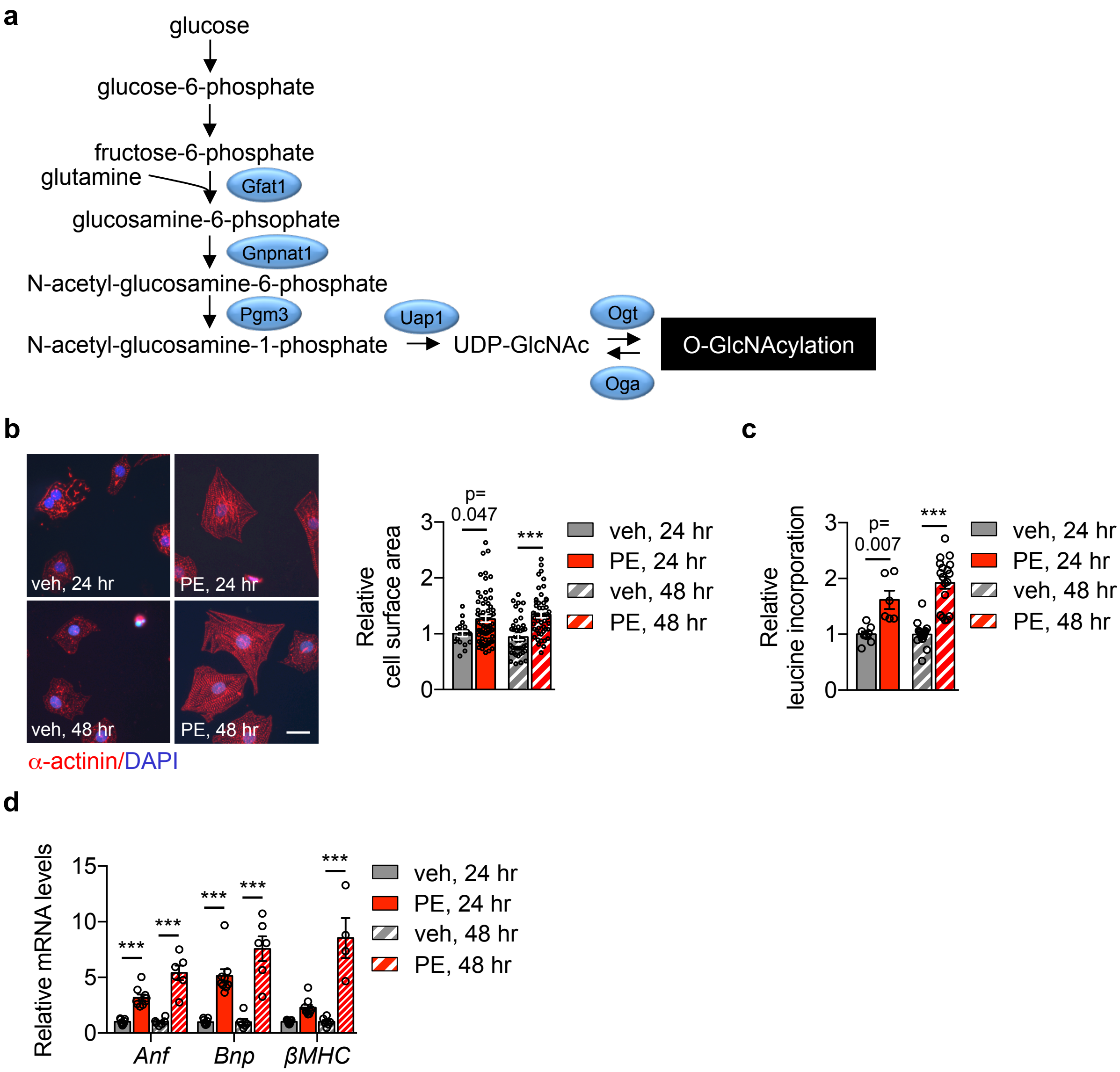

Supplementary Figure 1. Phenylephrine induces hypertrophic growth in cardiac myocytes.

a. The hexosamine biosynthetic pathway (HBP).

b. NRVMs in culture were treated with phenylephrine (PE, 50 μM) for 24 h and 48 h, respectively. Representative images of NRVMs stained with α-actinin antibody (red) and DAPI (blue) are shown. Scale: 20 μm. Bar graph depicts relative fold changes in cell surface area normalized to the vehicle (veh, 24 h) group. N = 19 cells for veh/24 hr; n = 66 cells for PE/24 hr; n = 43 cells for veh/48 hr; n = 50 cells for PE/48 hr. At least 3 independent experiments were conducted with 2-3 samples/group/experiment.

c. Quantification of leucine incorporation in NRVMs showed significant upregulation of protein synthesis by PE treatment. N = 6 for veh/24 hr; n = 6 for PE/24 hr; n = 19 for veh/48 hr; n = 18 for PE/48 hr.

d. Cardiomyocyte hypertrophic marker genes (*Anf*, *Bnp*, *βMHC*) were strongly induced by PE in NRVMs as determined by real-time PCR. N = 4-9. Data are shown as mean ± SEM. Significance was calculated by two-way ANOVA, followed by Tukey's test. ***, $p < 0.001$. Source data are provided as a Source Data file.

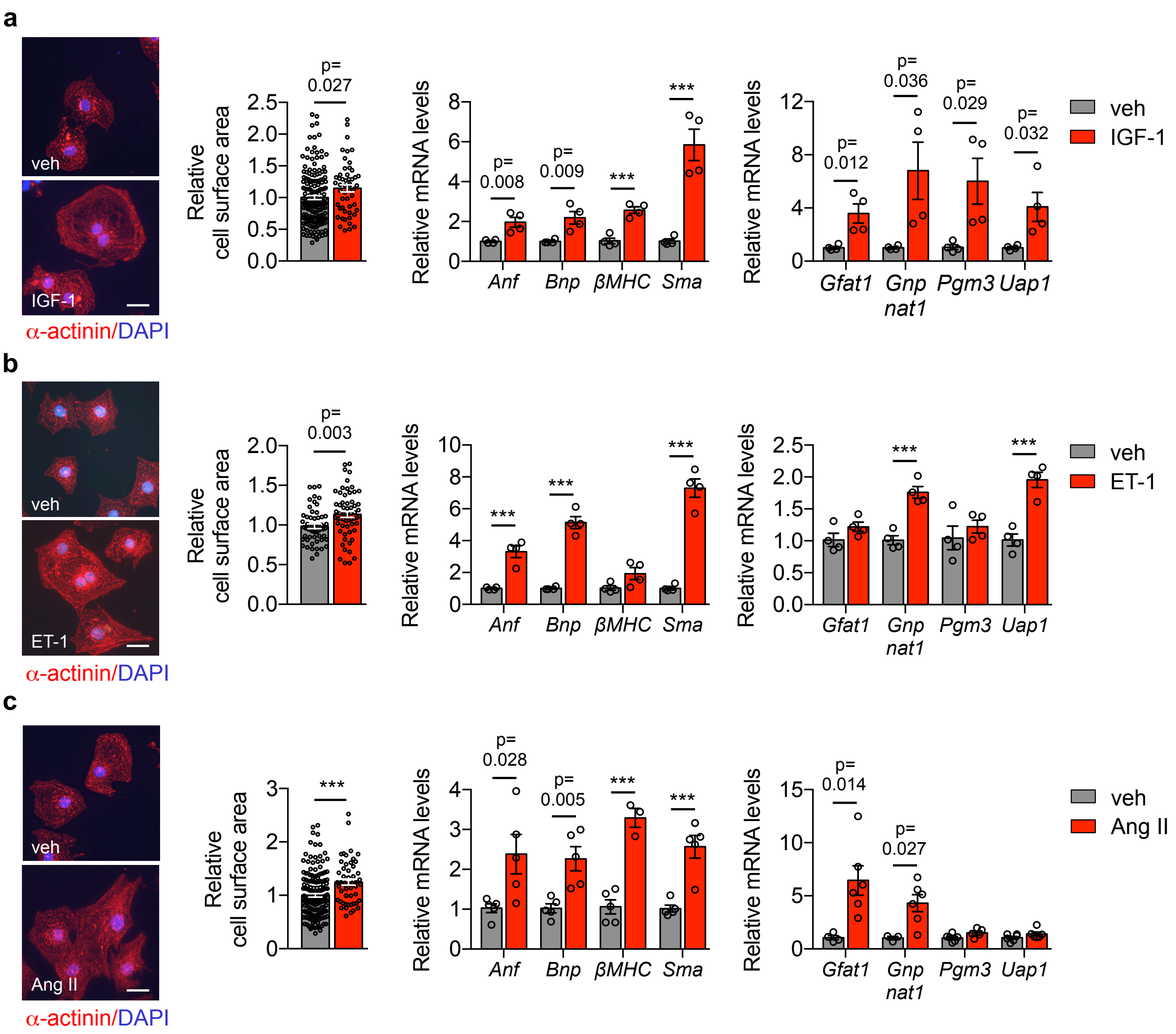

Supplementary Figure 2. Induction of the HBP by hypertrophic stimuli in cardiac myocytes.

- **a.** NRVMs in culture were treated with IGF-1 (10 nM) for 24 h. Cardiomyocyte staining for α-actinin showed a significant increase in cell size. Scale: 20 μM. N = 174 cells for veh; n = 52 cells for IGF-1. At least 3 independent experiments were conducted with 2-3 samples/group/experiment. Genes of both hypertrophic markers and the HBP were strongly induced. N = 4.
- **b.** Endothelin-1 (ET-1, 10 nM) treatment in NRVMs for 24 h stimulated cell growth (N = 54 cells for veh and n = 60 for ET-1 groups), which was associated with elevation in gene expression of both hypertrophic markers and the HBP. At least 3 independent experiments were conducted with 2-3 samples/group/experiment for cell size quantification. Scale: 20 μM. N = 4.
- **c.** Angiotensin II (Ang II, 1 μM, 24 h) administration led to a strong increase of cardiomyocyte size (N = 174 cells for veh and n = 52 cells for Ang II groups). At least 3 independent experiments were conducted with 2-3 samples/group/experiment. Gene of both hypertrophic markers and the HBP were significantly upregulated. Scale: 20 μM. N = 3-6. Data are shown

as mean ± SEM. Significance was calculated by Student's t test (two-tailed). ***, $p < 0.001$. Source data are provided as a Source Data file.

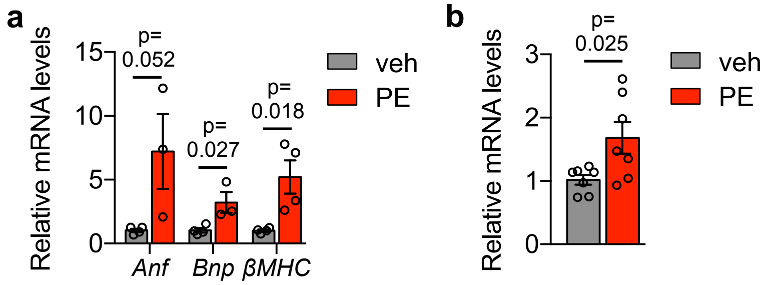

Supplementary Figure 3. Induction of the HBP by hypertrophic stimuli in adult cardiac myocytes.

a. Adult cardiomyocytes were isolated from 8-week old wild type mice. PE (50 µM) treatment was conducted for 24 h and total mRNA was extracted. Gene expression of hypertrophic markers was determined by qPCR. N = 3-4 per group.

b. *Gfat1* mRNA expression was assessed by qPCR. N = 7 for each group. Data are shown as mean ± SEM. Significance was calculated with Student's t test (two-tailed). Source data are provided as a Source Data file.

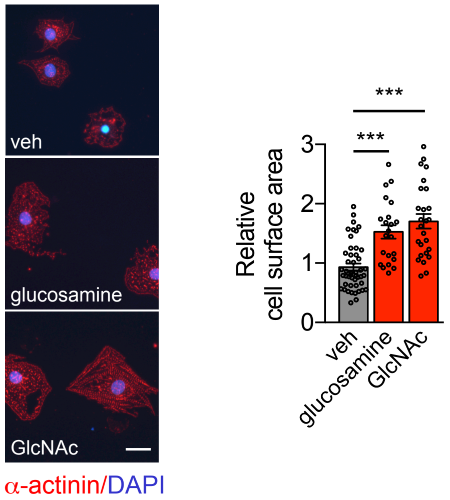

Supplementary Figure 4. Supplementation of HBP products is sufficient to induce hypertrophy in cardiac myocytes.

NRVMs in culture were treated with either glucosamine (50 µM) or GlcNAc (5 mM) for 48 h. Representative images of NRVMs stained with α-actinin antibody (red) and DAPI (blue) are shown. Scale: 20 µM. Bar graph at right depicts relative fold changes in cell surface area normalized to the vehicle (veh) group. N = 47 cells for veh; n = 22 cells for glucosamine; n = 26 cells for GlcNAc. At least 3 independent experiments were conducted with 2-3 samples/group/experiment. Data are presented as mean ± SEM. Significance was calculated with Student's t test (two-tailed). ***, $p < 0.001$. Source data are provided as a Source Data file.

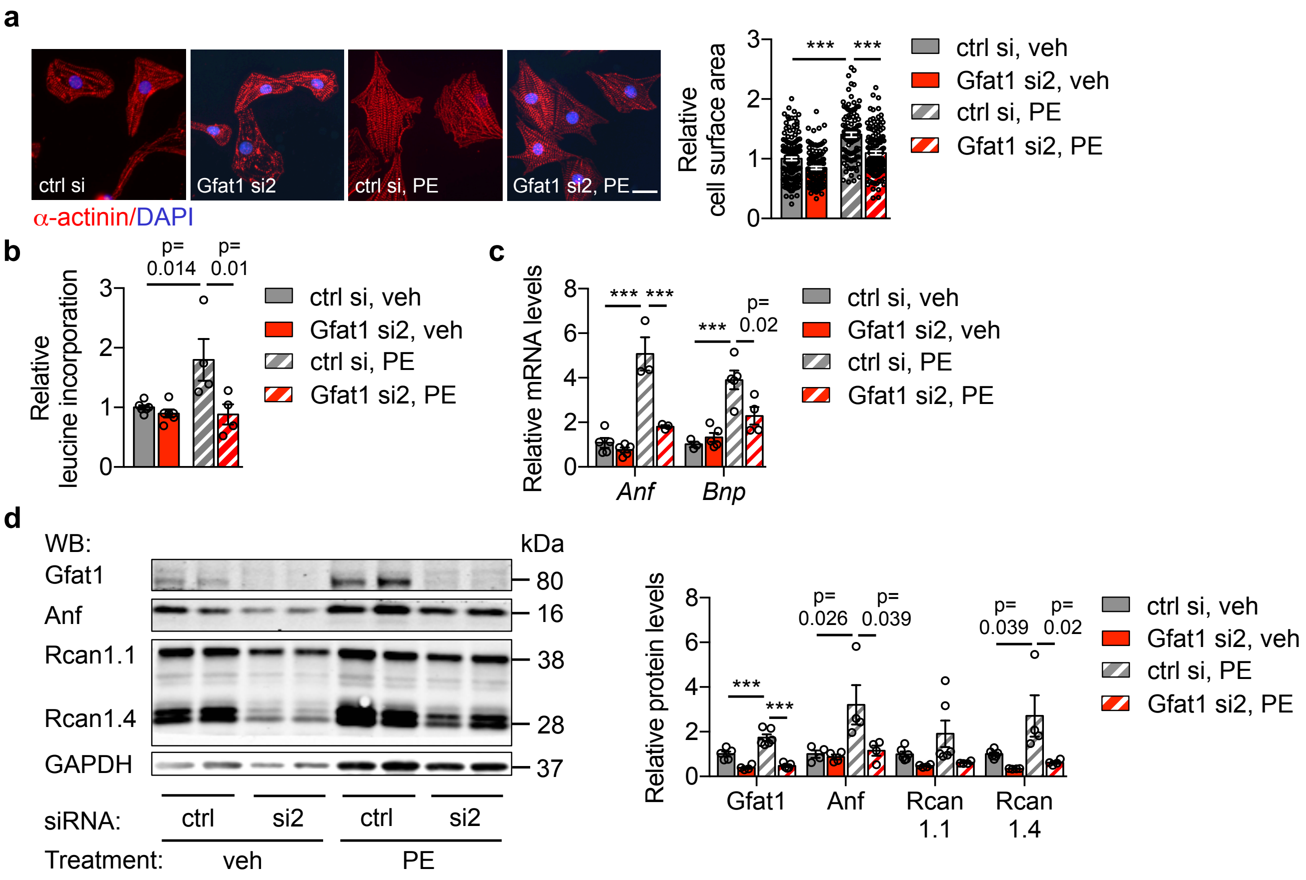

Supplementary Figure 5. Gfat1 is required for hypertrophic growth in cardiomyocytes.

- **a.** NRVMs were first transfected with either control siRNA (ctrl si) or Gfat1 siRNA-#2 (Gfat1 si2) and subsequently treated with PE for 48 h. Gfat1 si2 is independent of Gfat1 si1 used in Figure 3. Representative images of NRVMs stained with α-actinin antibody (red) and DAPI (blue) (left) are shown here. Scale: 20 μM. Quantification at right indicates a significant decrease in cell size after Gfat1 silencing. N = 160 cells for ctrl si/veh; n = 112 cells for Gfat1 si2/veh; n = 102 cells for ctrl si/PE; n = 148 cells for Gfat1 si/PE. At least 3 independent experiments were conducted with 2-3 samples/group/experiment.
- **b.** Leucine incorporation assay showed strong suppression of protein synthesis after Gfat1 knockdown by si2. N = 6 for veh; n = 4 for PE.
- **c.** Quantification of the mRNA level of hypertrophic marker genes (*Anf* and *Bnp*) after normalization to the ctrl si/veh group. N = 3-5.
- **d.** Representative immunoblots of Gfat1, Rcan1.1, Rcan1.4, and Anf after PE treatment are shown. Quantification is at the right. N = 4-6. Data are shown as mean ± SEM. Significance was calculated by two-way ANOVA, followed by Tukey's test. ***, $p < 0.001$. Source data are provided as a Source Data file.

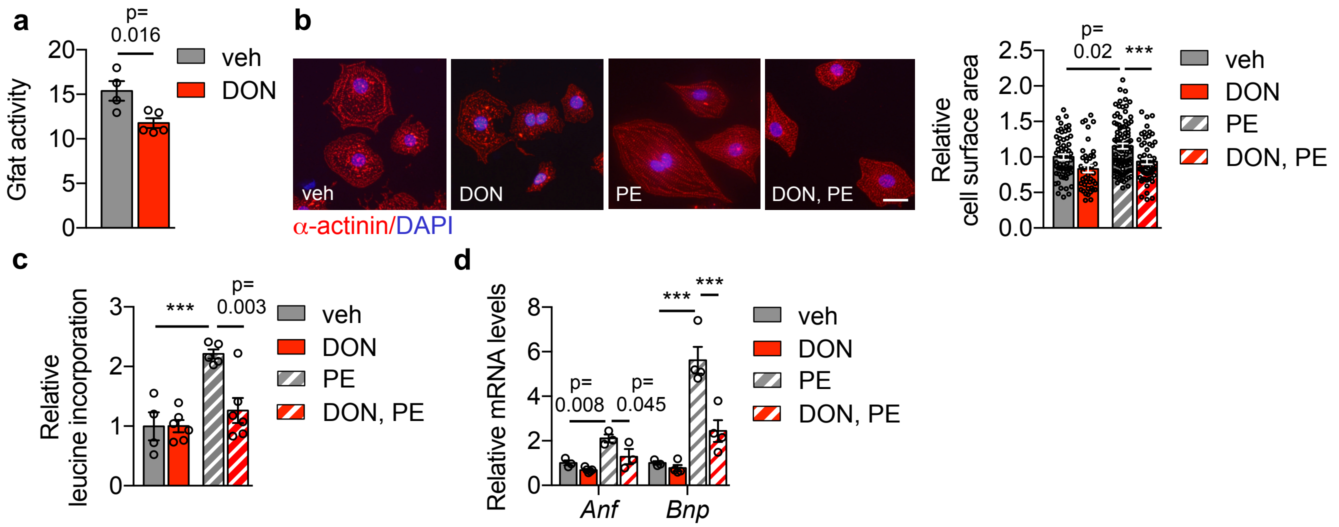

Supplementary Figure 6. Gfat1 inhibition attenuates hypertrophic growth in cardiac myocytes.

a. NRVMs were treated with DON (20 mM), a Gfat1 inhibitor, for 48 h. Cardiomyocyte proteins were extracted for Gfat enzymatic assay. The Gfat activity was presented by nmol glutamate/mg protein/minute. Student's t test (two-tailed) was used to calculate the significance. N = 4 for veh; n = 5 for DON.

b. NRVMs were treated with or without PE in the presence or absence of DON for 48 h. Cells were then stained with α-actinin antibody (red) and DAPI (blue) (left). Scale: 20 μM. Gfat1 inhibition led to a significant decrease in hypertrophic growth as shown by cell size quantification (right). N = 62 cells for veh; n = 42 cells for DON; n = 95 cells for PE; n = 61 cells for DON/PE. At least 3 independent experiments were conducted with 2-3 samples/group/experiment.

c. Protein synthesis was greatly diminished by Gfat1 inhibition as examined by leucine incorporation. N = 4 for veh; n = 5 for PE; n = 6 for all other groups.

d. Induction of hypertrophic marker genes by PE was reduced at the mRNA level in the presence of Gfat1 inhibitor DON. N = 3-5. Data are shown as mean ± SEM. Significance was calculated by two-way ANOVA, followed by Tukey's test. ***, $p < 0.001$. Source data are provided as a Source Data file.

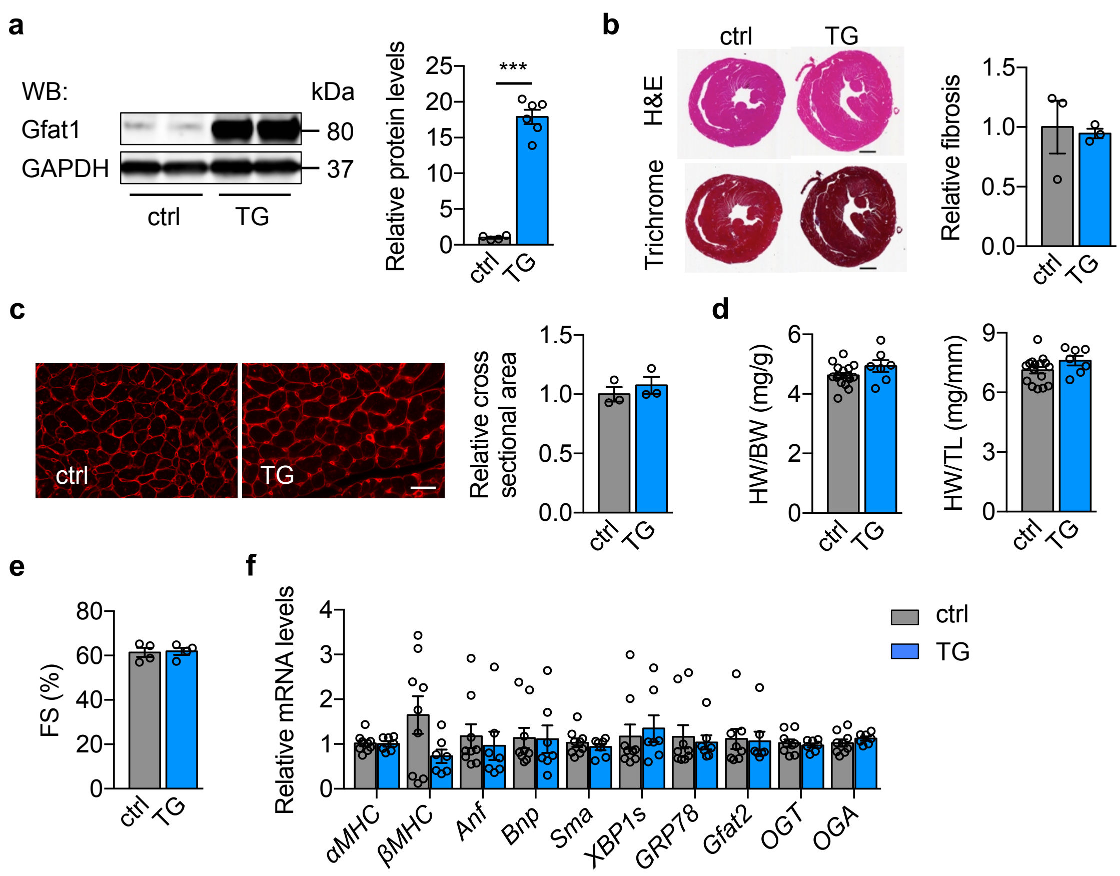

Supplementary Figure 7. The inducible Gfat1 transgenic mouse model.

- **a.** Gfat1 protein was significantly increased in the transgenic heart. Gfat1 transgenic (TG) mice along with controls were placed on regular drinking water for 2 weeks to induce Gfat1 gene expression. Cardiac tissues were harvested for immunoblotting. N = 4 for ctrl; n = 6 for TG.
- **b.** Gfat1 TG hearts did not show appreciable changes at the histological level. Fibrosis was not altered. Scale: 1 mm. N = 3.
- **c.** Gfat1 overexpression in the heart did not affect cardiomyocyte size. Heart sections were used for wheat germ agglutinin (WGA) staining and cell cross-sectional area was measured by Image J. Scale: 20 μm. N = 3.
- **d.** The Gfat1 transgenic mice did not show appreciable differences in heart weight/body weight ratio (HW/BW) or heart weight/tibia length ratio (HW/TL) at baseline. N = 16 for ctrl; n = 7 for TG.
- **e.** Cardiac function was maintained in Gfat1 transgenic mice at baseline as assayed by echocardiography for fractional shortening (FS). N = 4.
- **f.** No differences were detected in the transcriptional profiles of genes involved in cardiac hypertrophy *(αMHC*, *βMHC*, *Anf*, *Bnp*, *Sma*), unfolded protein response (*XBP1s*, *GRP78*), and HBP (*Gfat2*, *OGT*, *OGA*) between control and TG mice at the basal level. N = 9 for ctrl; n = 7 for TG. Data are shown as mean ± SEM. Student's t test (two-tailed) was conducted. ***, $p < 0.001$. Source data are provided as a Source Data file.

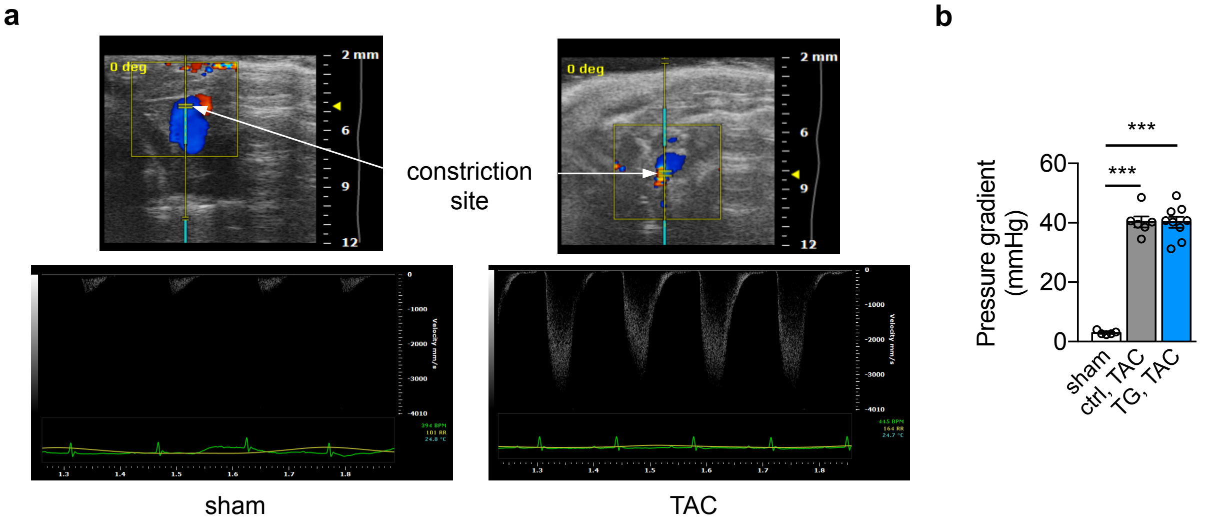

Supplementary Figure 8. Thoracic aortic constriction (TAC) in control and Gfat1 transgenic mice.

a. After sham or TAC surgery for 24 h, Doppler imaging was conducted to measure peak aortic velocity across the constriction site. Representative images were shown for sham and TAC operations, respectively.

b. Pressure gradient was calculated by $4 \times V_{max}^2$, according to the modified Bernoulli equation. N = 5 for sham; n = 6 for ctrl/TAC; n = 9 for TG/TAC. Data are shown as mean ± SEM. Student's t test (two-tailed) was conducted. ***, $p < 0.001$. Source data are provided as a Source Data file.

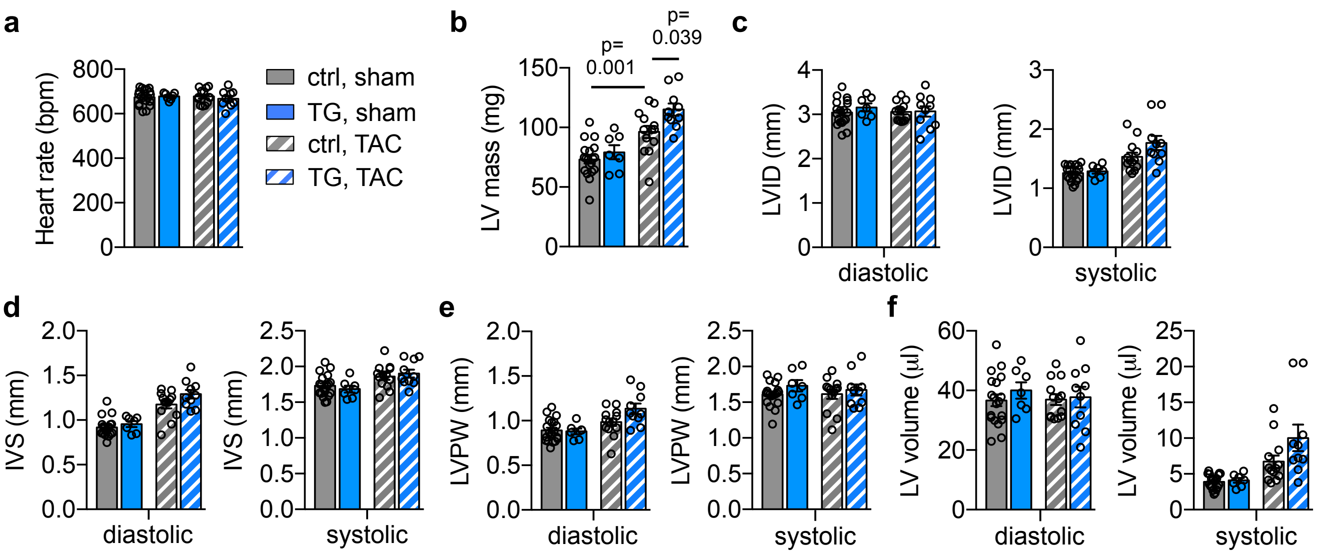

Supplementary Figure 9. Gfat1 overexpression exacerbates cardiac response by pressure overload.

- **a.** Cardiac-specific Gfat1 overexpression mice (TG) and the littermate controls (ctrl) were subjected to sham or thoracic aortic constriction (TAC). Heart function was determined by echocardiography at 3 weeks post-surgery. Heart rate was not affected. N = 19 for ctrl/sham; n = 7 for TG/sham; n = 13 for ctrl/TAC; n = 10 for TG/TAC.
- **b.** TAC led to a significant increase in left ventricle (LV) mass in control animals, which was further augmented in the TG mice. N = 19 for ctrl/sham; n = 7 for TG/sham; n = 13 for ctrl/TAC; n = 10 for TG/TAC.
- **c.** Gfat1 overexpression in the heart caused a trend of elevated response in left ventricular internal diameter (LVID) at systole. N = 19 for ctrl/sham; n = 7 for TG/sham; n = 13 for ctrl/TAC; n = 10 for TG/TAC.
- **d.** No significant difference in interventricular septum (IVS) was observed at either diastole or systole. N = 19 for ctrl/sham; n = 7 for TG/sham; n = 13 for ctrl/TAC; n = 10 for TG/TAC.
- **e.** LV posterior wall (LVPW) diameter did not differ between control and TG mice. N = 19 for ctrl/sham; n = 7 for TG/sham; n = 13 for ctrl/TAC; n = 10 for TG/TAC.
- **f.** Consistent with the exacerbated cardiac response by pressure overload, Gfat1 overexpression in TG mice led to a trend of increased LV volume compared to control animals after TAC. N = 19 for ctrl/sham; n = 7 for TG/sham; n = 13 for ctrl/TAC; n = 10 for TG/TAC. Data are shown as mean ± SEM. Significance was calculated by two-way ANOVA, followed by Tukey's test. Source data are provided as a Source Data file.

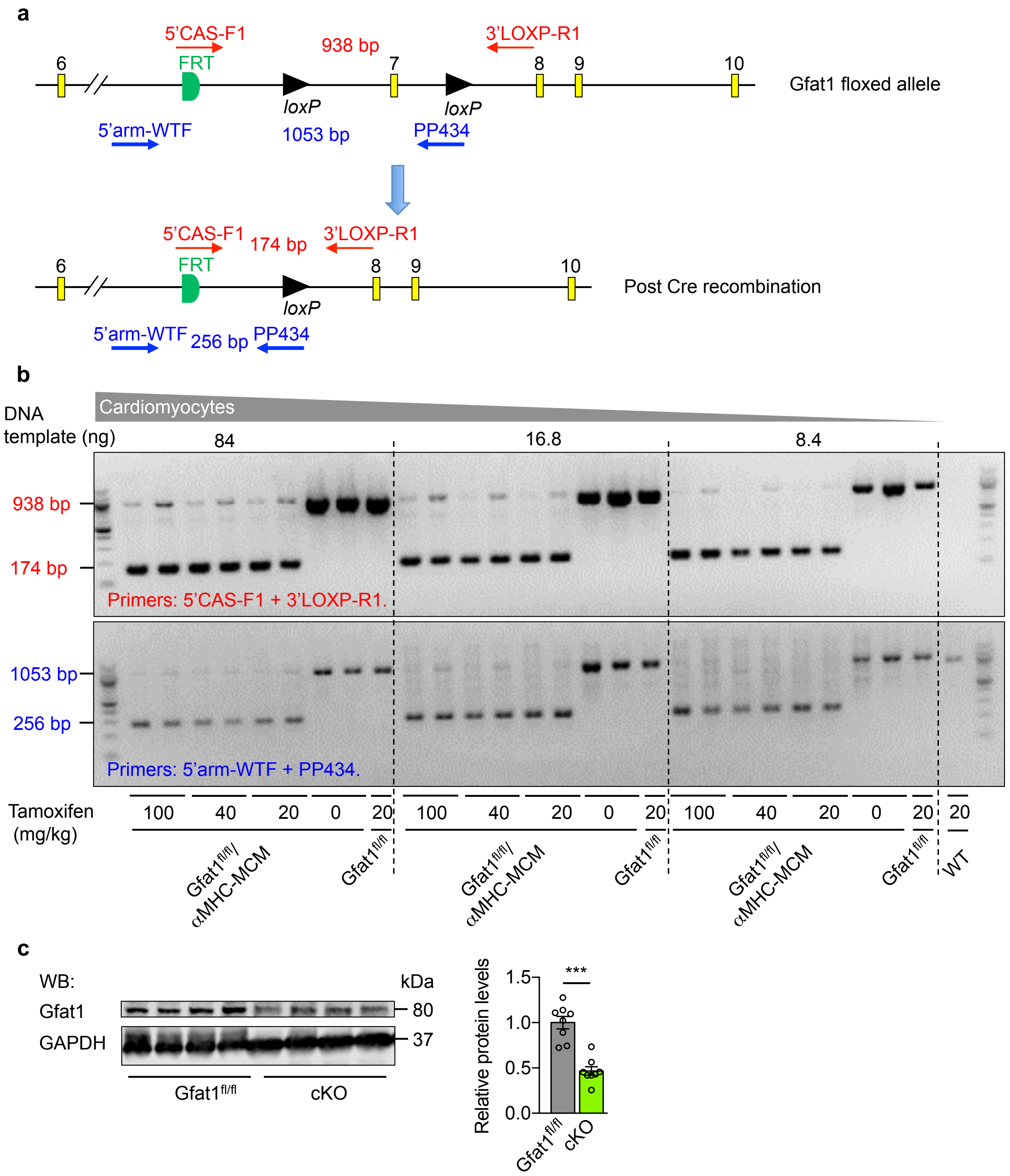

Supplementary Figure 10. The inducible Gfat1 conditional knockout mouse model.

- **a.** The scheme of cardiomyocyte-specific deletion of *Gfat1*. The two pairs of primers are shown.
- **b.** Cardiomyocytes were isolated from adult mice after various tamoxifen treatments for 5 consecutive days. Genomic DNA was purified and diluted. PCR was conducted with two pairs of primers in **a**. Note that PCR products were bigger in pre-recombination genomic DNA. After Cre

excision, the PCR products were smaller, indicating deletion of exon 7. Experiments were done with 1-2 biological replicates in a dose-dependent manner.

c. Gfat1 protein expression was reduced in the cKO mouse hearts. Cardiac tissues were collected from 3-4 months old animals and used for Western blotting. N = 8. Data are shown as mean ± SEM. Student's t test (two-tailed) was conducted. ***, $p < 0.001$. Source data are provided as a Source Data file.

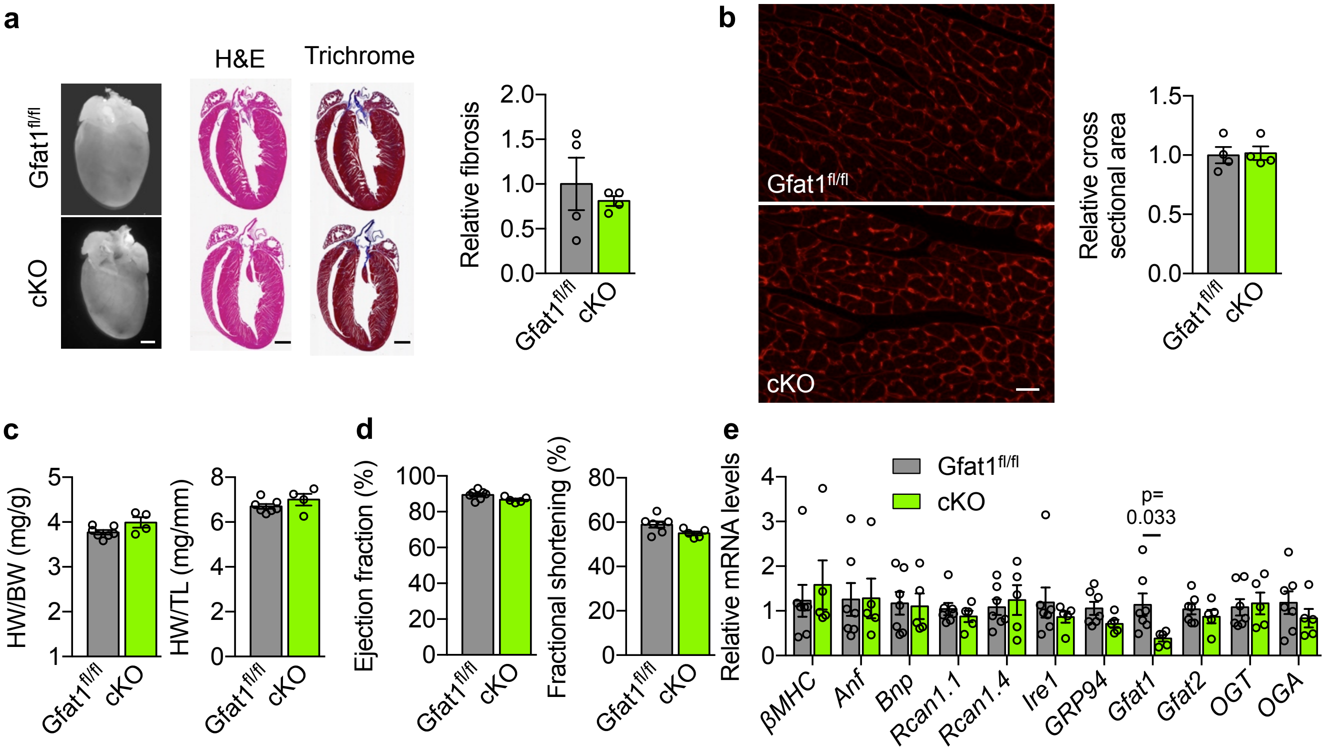

Supplementary Figure 11. Gfat1 cardiac-specific conditional knockout mouse model.

a. Deletion of *Gfat1* from the heart (cKO) did not cause changes in heart histology at baseline, as shown by hematoxylin & eosin and Masson's trichrome staining. No appreciable changes in fibrosis were found. Scale: 1 mm. N = 4.

b. Cardiomyocyte cross sectional area did not differ between control and cKO mice at the basal level. WGA staining was conducted and quantification was done with Image J. Scale: 20 μm. N = 4.

c. Gfat1 deficiency did not lead to cardiac hypertrophic growth at baseline as shown by insignificant changes in heart weight/body weight ratio (HW/BW) and heart weight/tibia length ratio (HW/TL). N = 7 for Gfat1$^{fl/fl}$; n = 4 for cKO.

d. No change in cardiac performance was noticed for either ejection fraction or fractional shortening as examined by echocardiography at baseline. N = 7 for Gfat1$^{fl/fl}$; n = 5 for cKO.

e. No transcriptional changes in hypertrophic genes (*βMHC*, *Anf*, *Bnp*, *Rcan1.1*, *Rcan1.4*), unfolded protein response (*Ire1*, *GRP94*), and HBP (*Gfat2*, *OGT*, *OGA*) were detected between control and cKO mice. N = 7 for Gfat1$^{fl/fl}$; n = 5 for cKO. Data are shown as mean ± SEM. Student's t test (two-tailed) was conducted. Source data are provided as a Source Data file.

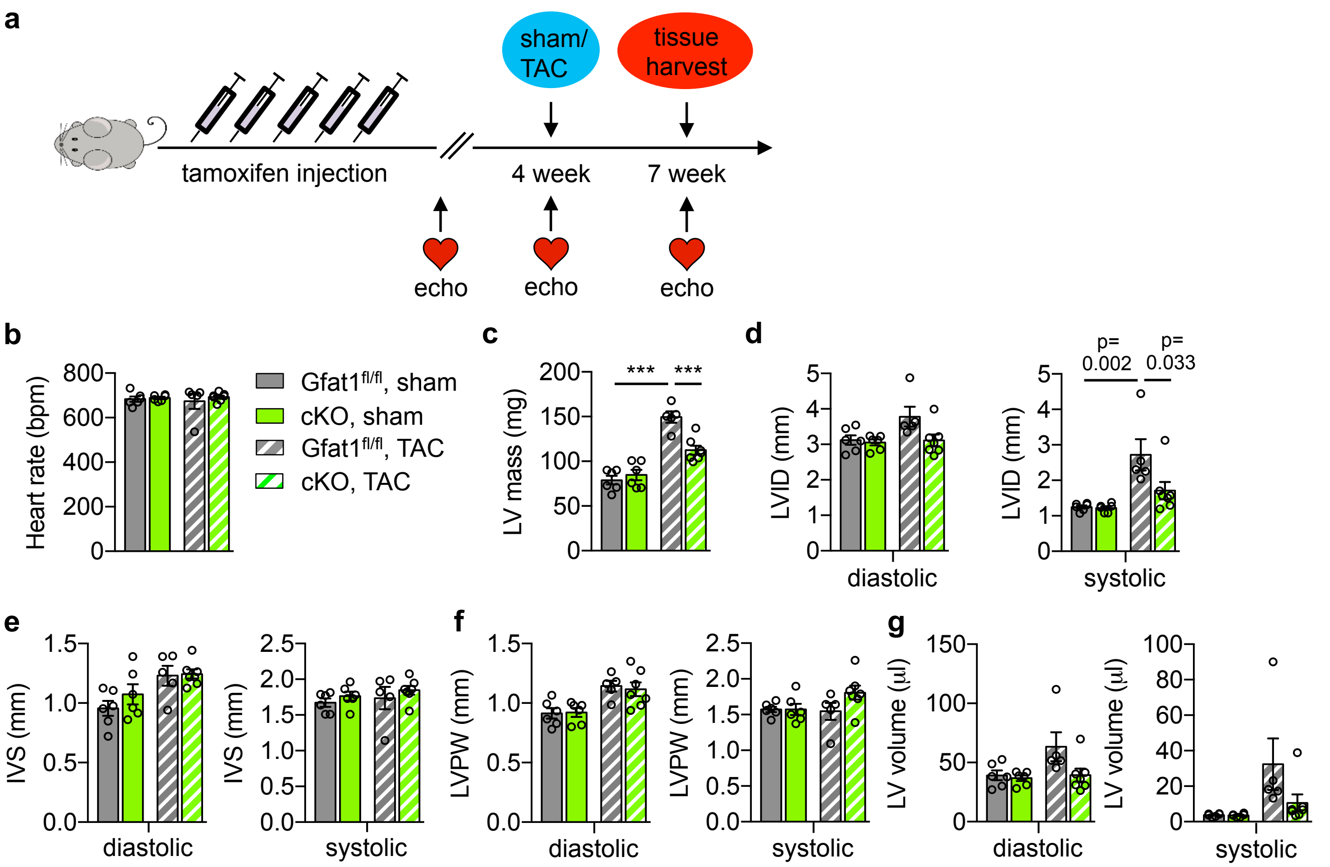

Supplementary Figure 12. Gfat1 deficiency reduces pathological hypertrophy and improves cardiac dysfunction in response to pressure overload.

a. Schematic representation of the experimental design for tamoxifen injection, echocardiography, surgery, and tissue harvest. Mice were subjected to surgery (sham or TAC) when cardiac function was recovered at 4 weeks post-tamoxifen injection.

b. No change in heart rate was observed between control and cKO mice, as shown by echocardiography. N = 6 for Gfat1$^{fl/fl}$/sham; n = 6 for cKO/sham; n = 5 for Gfat1$^{fl/fl}$/TAC; n = 7 for cKO/TAC.

c. The cKO mice displayed less LV mass, which is consistent with reduced pathological cardiac remodeling. N = 6 for Gfat1$^{fl/fl}$/sham; n = 6 for cKO/sham; n = 5 for Gfat1$^{fl/fl}$/TAC; n = 7 for cKO/TAC.

d. LVID at systole was decreased, suggesting improved cardiac systolic function. N = 6 for Gfat1$^{fl/fl}$/sham; n = 6 for cKO/sham; n = 5 for Gfat1$^{fl/fl}$/TAC; n = 7 for cKO/TAC.

e. IVS did not differ between control and cKO in either sham or TAC animals. N = 6 for Gfat1$^{fl/fl}$/sham; n = 6 for cKO/sham; n = 5 for Gfat1$^{fl/fl}$/TAC; n = 7 for cKO/TAC.

f. LVPW was not altered at either diastole or systole. N = 6 for Gfat1$^{fl/fl}$/sham; n = 6 for cKO/sham; n = 5 for Gfat1$^{fl/fl}$/TAC; n = 7 for cKO/TAC.

g. LV volume showed a trend of decreasing in cKO mice after TAC, which is consistent with improvements in cardiac function and performance. N = 6 for Gfat1$^{fl/fl}$/sham; n = 6 for cKO/sham; n = 5 for Gfat1$^{fl/fl}$/TAC; n = 7 for cKO/TAC. Data are shown as mean ± SEM. Significance was calculated by two-way ANOVA, followed by Tukey's test. ***, $p < 0.001$. Source data are provided as a Source Data file.

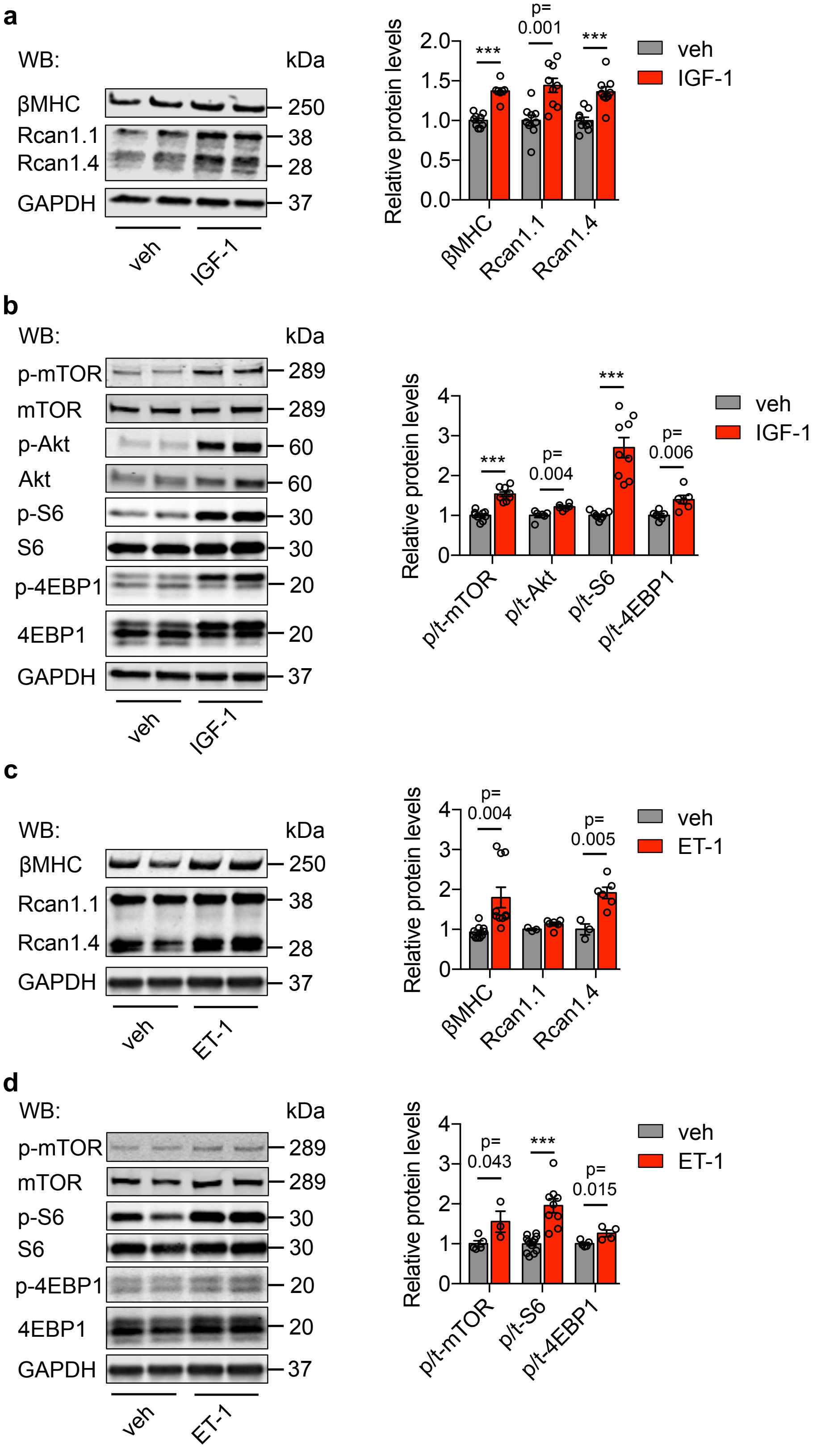

Supplementary Figure 13. Induction of the mTOR signaling by hypertrophic stimuli in cardiac myocytes.

a. NRVMs in culture were treated with IGF-1 for 24 h, thereby inducing genes of hypertrophic markers at the protein levels. N = 7 for βMHC/IGF-1; n = 9 for all other groups.

b. The mTOR signaling was induced by IGF-1 in NRVMs. N = 9 for p/t-mTOR and p/t-S6; n = 6 for p/t-Akt and p/t-4EBP1.

c. ET-1 treatment in NRVMs increased hypertrophic marker genes at the protein levels. N = 3-10.

d. ET-1 increased phosphorylation of mTOR and downstream effectors of the mTOR pathway. N = 3-13. Data are shown as mean ± SEM. Student's t test (two-tailed) was conducted. ***, $p < 0.001$. Source data are provided as a Source Data file.

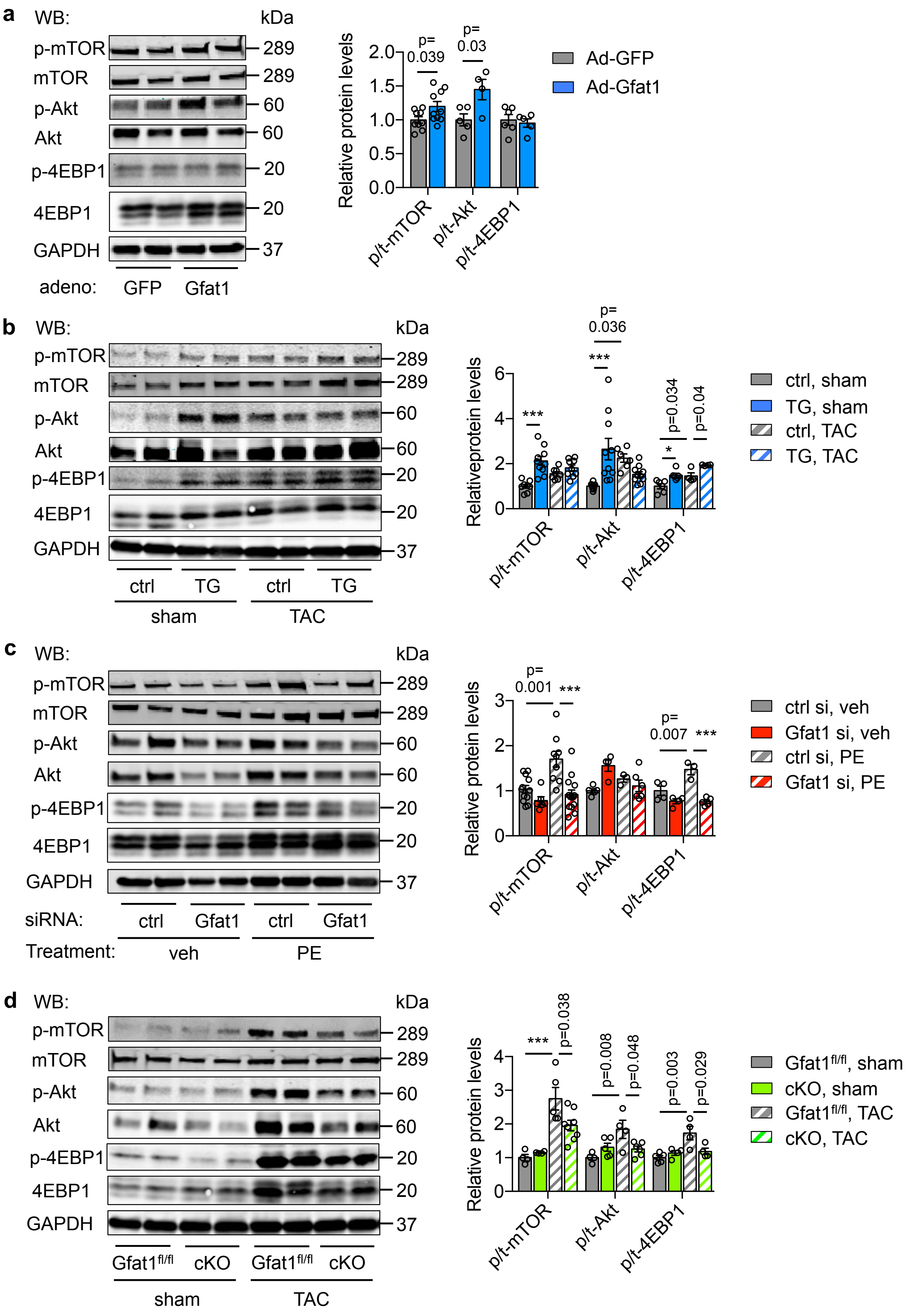
a
WB:
kDa
p-mTOR
mTOR
p-Akt
Akt
p-4EBP1
4EBP1
GAPDH
289
289
60
60
20
20
37
adeno:
GFP
Gfat1
Relative protein levels
2.0
1.5
1.0
0.5
0.0
p=
0.039
p=
0.03
p/t-mTOR
p/t-Akt
p/t-4EBP1
Ad-GFP
Ad-Gfat1
b
WB:
kDa
p-mTOR
mTOR
p-Akt
Akt
p-4EBP1
4EBP1
GAPDH
289
289
60
60
20
20
37
ctrl
TG
ctrl
TG
sham
TAC
Relativeprotein levels
6
4
2
0
***
***
p=
0.036
*
p=0.034
p=0.04
p/t-mTOR
p/t-Akt
p/t-4EBP1
ctrl, sham
TG, sham
ctrl, TAC
TG, TAC
c
WB:
kDa
p-mTOR
mTOR
p-Akt
Akt
p-4EBP1
4EBP1
GAPDH
289
289
60
60
20
20
37
siRNA:
ctrl
Gfat1
ctrl
Gfat1
Treatment:
veh
PE
Relative protein levels
3
2
1
0
p=
0.001
***
p=
0.007
***
p/t-mTOR
p/t-Akt
p/t-4EBP1
ctrl si, veh
Gfat1 si, veh
ctrl si, PE
Gfat1 si, PE
d
WB:
kDa
p-mTOR
mTOR
p-Akt
Akt
p-4EBP1
4EBP1
GAPDH
289
289
60
60
20
20
37
Gfat1fl/fl
cKO
Gfat1fl/fl
cKO
sham
TAC
Relative protein levels
4
3
2
1
0
***
p=0.038
p=0.008
p=0.048
p=0.003
p=0.029
p/t-mTOR
p/t-Akt
p/t-4EBP1
Gfat1fl/fl, sham
cKO, sham
Gfat1fl/fl, TAC
cKO, TAC

Supplementary Figure 14. HBP stimulates the mTOR signaling.

a. Gfat1 overexpression in NRVMs was sufficient to activate the mTOR signaling. N = 4-10. Student's t test (two-tailed) was conducted.

b. Gfat1 overexpression in the heart led to activation of the mTOR pathway. Gfat1 expression was turned on in the double transgenic mice for 2 weeks. After TAC, the heart was harvested to assess mTOR signaling. N = 4-10.

c. Gfat1 silencing in NRVMs diminished the mTOR pathway after PE treatment. N = 3-14.

d. Cardiac-specific knockout of Gfat1 led to reduction in the mTOR signaling, as evidenced by decreases in the phosphorylation of mTOR, Akt, and 4EBP1. N = 4-8. Data are shown as mean ± SEM. Significance was calculated by two-way ANOVA, followed by Tukey's test. *, $p = 0.011$; ***, $p < 0.001$. Source data are provided as a Source Data file.

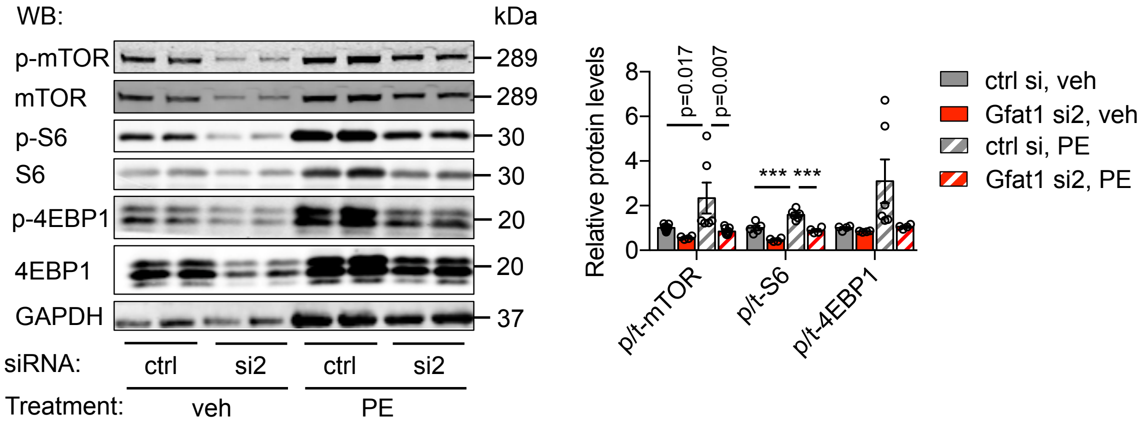

Supplementary Figure 15. Knockdown of Gfat1 inhibits PE-induced mTOR activation.

Gfat1 was decreased by siRNA-mediated knockdown. Here, an independent siRNA2 was used in comparison to the one used in Figure 7c and Supplementary Figure 14c. PE treatment was then conducted for 48 h. Protein extracts from NRVMs were used for immunoblotting of the mTOR pathway. N = 4-9. Data are shown as mean ± SEM. Significance was calculated by two-way ANOVA, followed by Tukey's test. ***, $p < 0.001$. Source data are provided as a Source Data file.

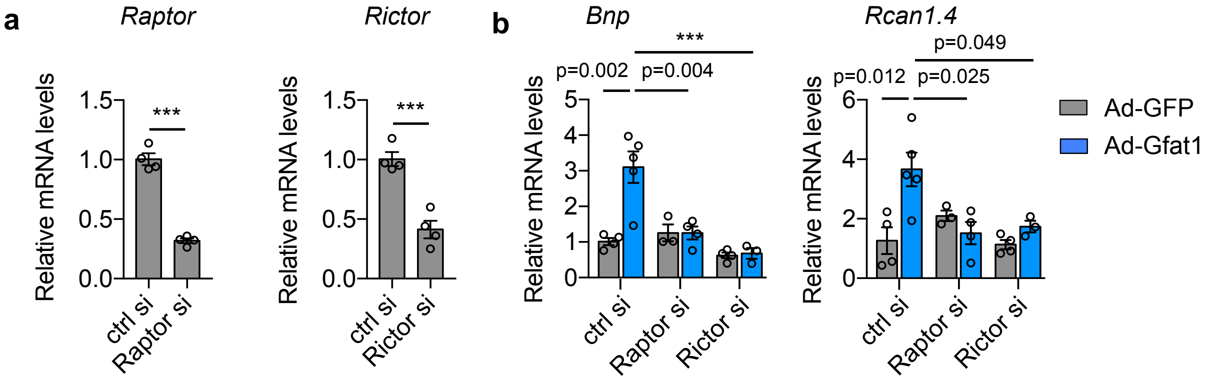

Supplementary Figure 16. Both mTORC1 and mTORC2 contribute to Gfat1-mediated cardiomyocyte growth.

a. The knockdown efficiency was evaluated by qPCR in NRVMs. N = 4. Student's t test (two-tailed) was conducted.

b. After knockdown of either Raptor or Rictor, adenovirus expressing GFP or Gfat1 was used to infect NRVMs. Total mRNA was isolated for qPCR analysis. Silencing of either Raptor or Rictor decreased Gfat1-mediated elevation of genes of the fetal gene program. N = 4 for Ad-GFP/ctrl si; n = 5 for Ad-Gfat1/ctrl si; n = 3 for Ad-GFP/Raptor si; n = 4 for Ad-Gfat1/Raptor si; n = 4 for Ad-GFP/Rictor si; n = 3 for Ad-Gfat1/Rictor si. Data are shown as mean ± SEM. Significance was calculated by two-way ANOVA, followed by Tukey's test. ***, $p < 0.001$. Source data are provided as a Source Data file.

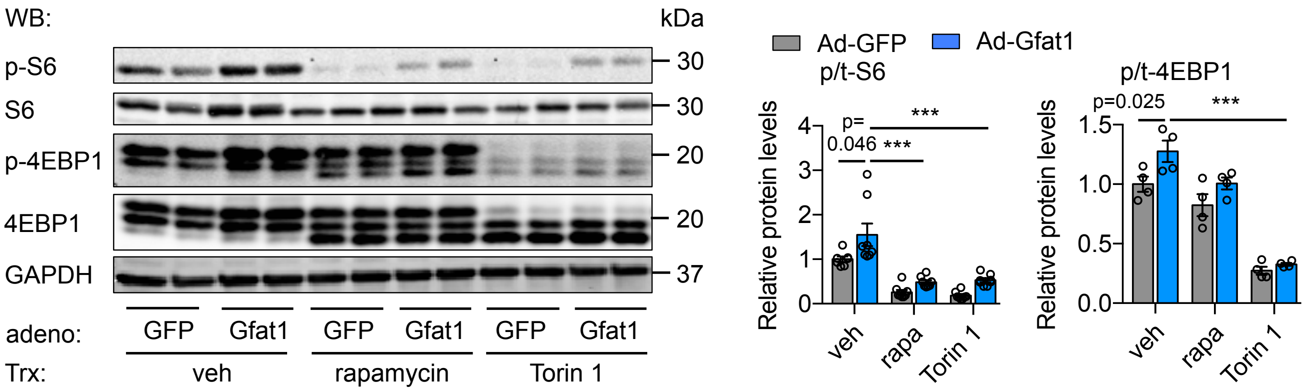

Supplementary Figure 17. Suppression of the mTOR signaling in NRVMs.

NRVMs were first infected by adenovirus expressing control GFP or Gfat1 for 24 h. Rapamycin (20 nM) or Torin 1 (50 nM) was then added to suppress the mTOR signaling. Immunoblotting was conducted to assess the inhibition efficiency. N = 8 for p/t-S6; n = 4 for p/t-4EBP1. Data are shown as mean ± SEM. Significance was calculated by two-way ANOVA, followed by Tukey's test. ***, $p < 0.001$. Source data are provided as a Source Data file.

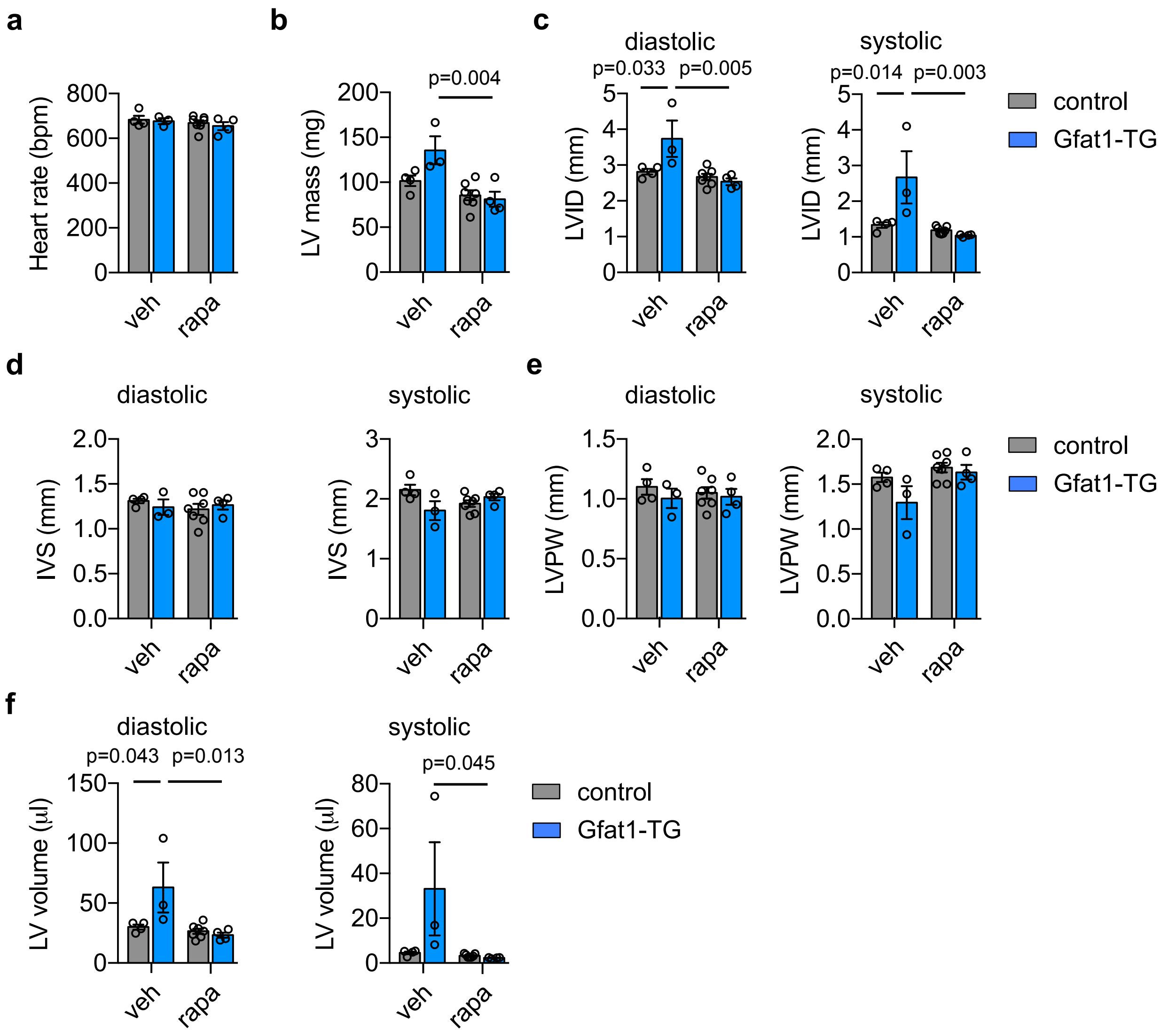

Supplementary Figure 18. Rapamycin treatment improves cardiac function in Gfat1 transgenic mice after pressure overload.

- **a.** No change in heart rate was observed between control and Gfat1 TG mice, as shown by echocardiography. Note that all mice underwent TAC surgery. N = 4 for control/veh; n = 3 for Gfat1-TG/veh; n = 7 for control/rapa; n = 4 for Gfat1-TG/rapa.
- **b.** The mice after rapamycin treatment displayed less LV mass, which is consistent with reduced pathological cardiac remodeling. N = 4 for control/veh; n = 3 for Gfat1-TG/veh; n = 7 for control/rapa; n = 4 for Gfat1-TG/rapa.
- **c.** LVID was decreased, suggesting improved cardiac systolic function. N = 4 for control/veh; n = 3 for Gfat1-TG/veh; n = 7 for control/rapa; n = 4 for Gfat1-TG/rapa.
- **d.** IVS did not differ between vehicle and rapamycin treatments. N = 4 for control/veh; n = 3 for Gfat1-TG/veh; n = 7 for control/rapa; n = 4 for Gfat1-TG/rapa.
- **e.** LVPW was not altered at either diastole or systole. N = 4 for control/veh; n = 3 for Gfat1-TG/veh; n = 7 for control/rapa; n = 4 for Gfat1-TG/rapa.
- **f.** LV volume showed a decrease after rapamycin treatment, which is consistent with improvements in cardiac function and performance. N = 4 for control/veh; n = 3 for Gfat1-TG/veh; n = 7 for

control/rapa; n = 4 for Gfat1-TG/rapa. Data are shown as mean ± SEM. Significance was calculated by two-way ANOVA, followed by Tukey's test. Source data are provided as a Source Data file.

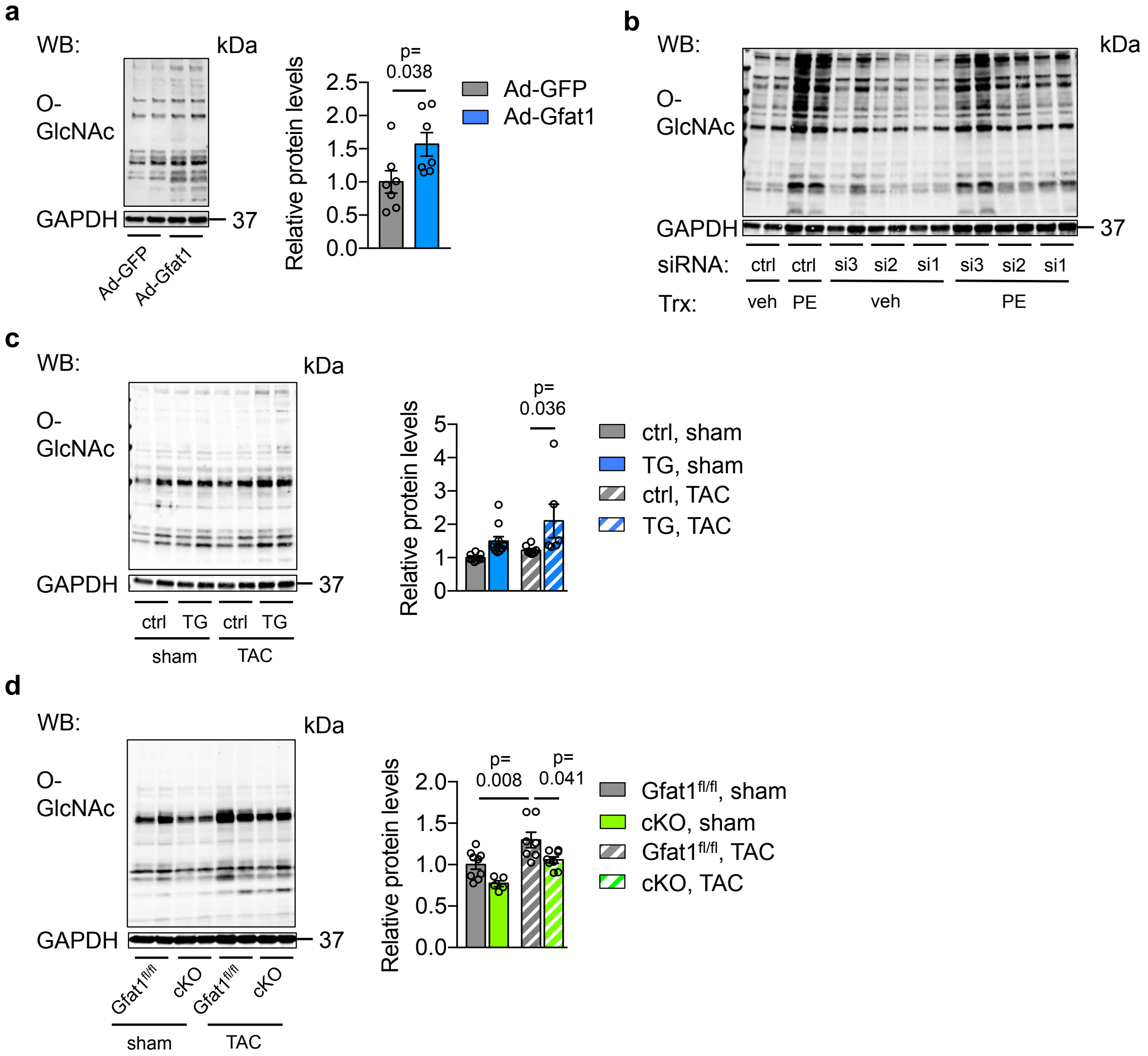

Supplementary Figure 19. Gfat1 expression is associated with elevation of protein O-GlcNAcylation.

- **a.** Gfat1 overexpression in NRVMs was sufficient to increase protein O-GlcNAcylation. N = 7. Student's t test (two-tailed) was conducted.
- **b.** Gfat1 silencing in NRVMs diminished O-GlcNAcylation after PE treatment. Three sequence-independent siRNA oligos were used to knockdown Gfat1. PE treatment was then conducted. Experiments were independently repeated for 3 times with similar results.
- **c.** Gfat1 overexpression in the heart led to an increase in O-GlcNAcylation. Gfat1 expression was turned on in the double transgenic mice for 2 weeks. After TAC, the heart was harvested to assess O-GlcNAcylation. N = 9 for ctrl/sham; n = 11 for TG/sham; n = 8 for ctrl/TAC; n = 6 for TG/TAC.

d. Cardiac-specific knockout of Gfat1 led to reduction in O-GlcNAcylation. N = 9 for Gfat1$^{fl/fl}$/sham; n = 5 for cKO/sham; n = 7 for Gfat1$^{fl/fl}$/TAC; n = 8 for cKO/TAC. Data are shown as mean ± SEM. Significance was calculated by two-way ANOVA, followed by Tukey's test. Source data are provided as a Source Data file.

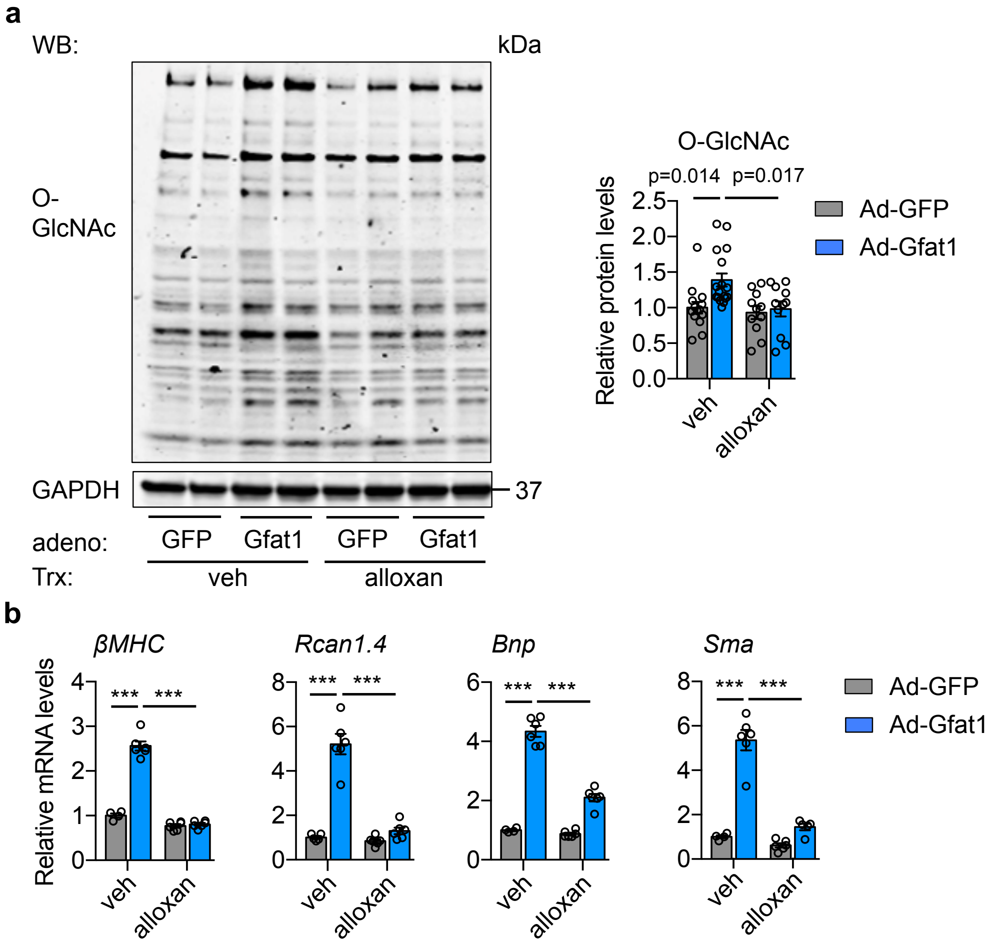

Supplementary Figure 20. Alloxan inhibits Gfat1-induced hypertrophic growth.

a. Alloxan treatment suppressed protein O-GlcNAcylation, as examined by Western blotting. N = 14 for Ad-GFP/veh; n = 17 for Ad-Gfat1/veh; n = 11 for Ad-GFP/Alloxan; n = 11 for Ad-Gfat1/Alloxan.

b. Inhibition of O-GlcNAcylation decreased cardiomyocyte size from Gfat1 overexpression. Real-time PCR was conducted to evaluate mRNA expression of the fetal gene program. N =4-6. Data are shown as mean ± SEM. Significance was calculated by two-way ANOVA, followed by Tukey's test. ***, p < 0.001. Source data are provided as a Source Data file.

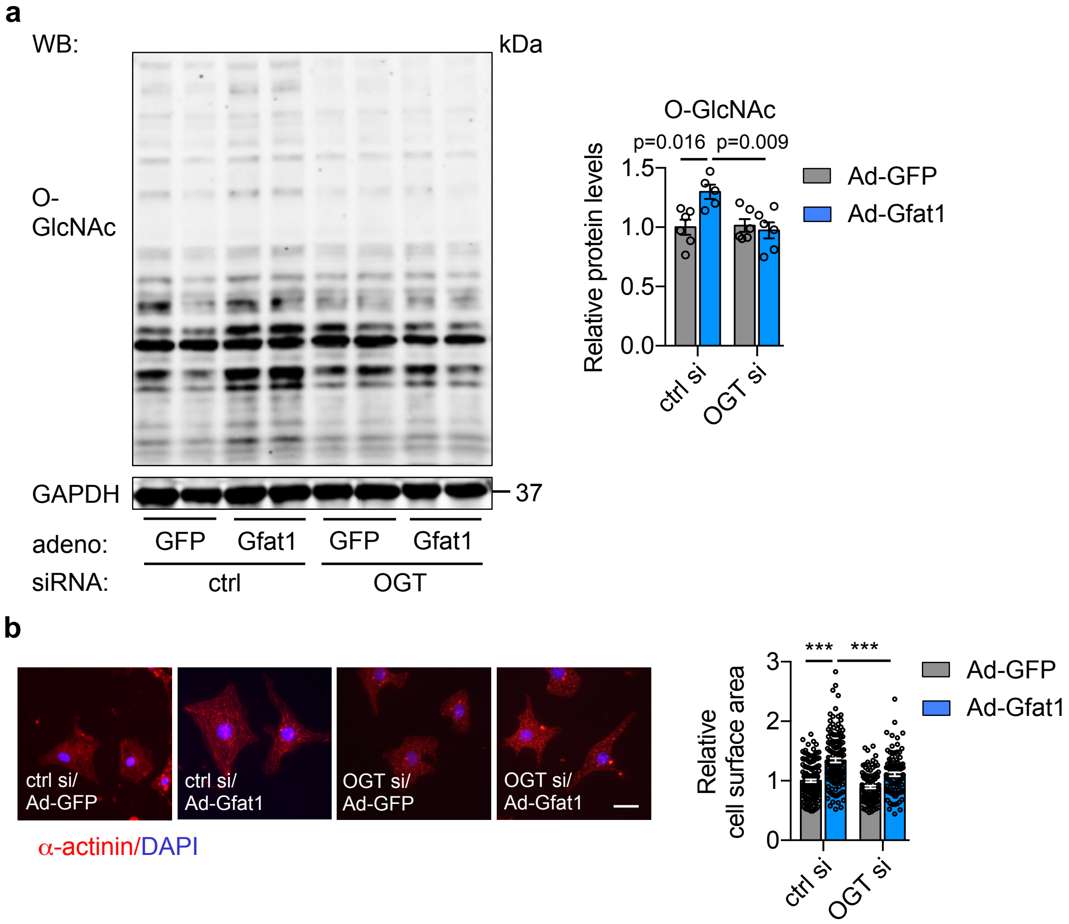

Supplementary Figure 21. OGT knockdown decreases Gfat1-induced hypertrophic growth.

- **a.** Knockdown of OGT in NRVMs led to a decrease in O-GlcNAcylation. N = 5 for ctrl si/Ad-Gfat1; n = 6 for all other groups.
- **b.** OGT silencing inhibited cardiomyocyte growth from Gfat1 overexpression. Scale: 20 μm. N = 143 cells Ad-GFP/ctrl si; n = 146 cells for Ad-Gfat1/ctrl si; n = 108 cells for Ad-GFP/OGT si; n = 104 cells for Ad-Gfat1/OGT si. At least 3 independent experiments were conducted with 2-3 samples/group/experiment. Data are shown as mean ± SEM. Significance was calculated by two-way ANOVA, followed by Tukey's test. ***, $p < 0.001$. Source data are provided as a Source Data file.

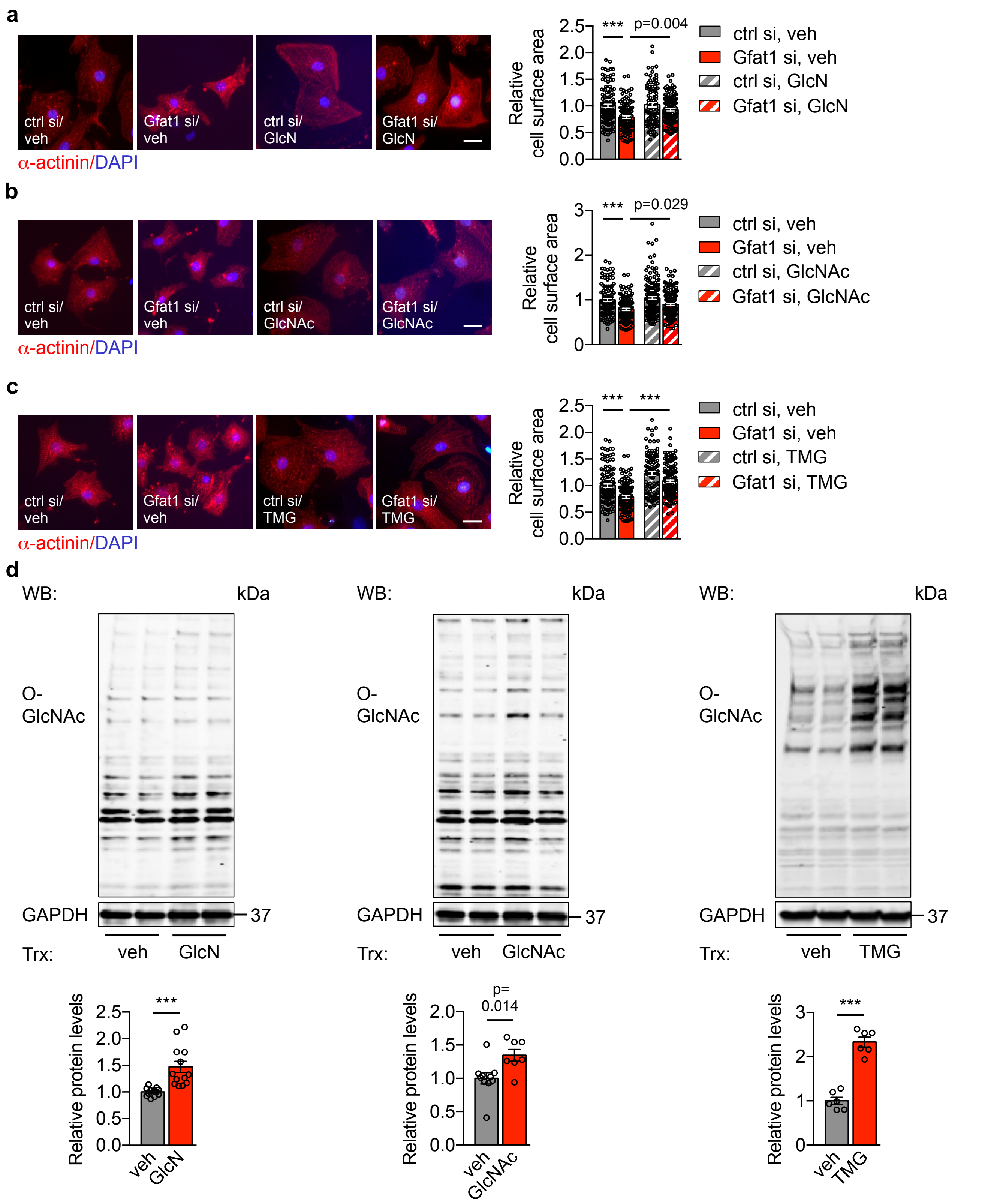

Supplementary Figure 22. Supplementation of the HBP products rescues cardiomyocyte growth from Gfat1 silencing.

a. NRVMs were transfected by siRNA against Gfat1. Glucosamine treatment was conducted for 24 h. Cardiomyocytes were stained for α-actinin and quantified by Image J. Scale: 20 μm. N = 119

cells for ctrl si/veh; n = 115 cells for Gfat1 si/veh; n = 91 cells for ctrl si/GlcN; n = 124 cells for Gfat1 si/GlcN. At least 3 independent experiments were conducted with 2-3 samples/group/experiment.

b. GlcNAc was used to treat NRVMs. Gfat1 was reduced by siRNA-mediated knockdown. Cardiomyocyte size was measured and compared. Scale: 20 μm. N = 119 cells for ctrl si/veh; n = 115 cells for Gfat1 si/veh; n = 158 cells for ctrl si/GlcNAc; n = 172 cells for Gfat1 si/GlcNAc. At least 3 independent experiments were conducted with 2-3 samples/group/experiment.

c. TMG (Thiamet-G) treatment led to an increase of O-GlcNAcylation and rescue of cardiomyocyte growth under the condition of Gfat1 silencing. Scale: 20 μm. N = 119 cells for ctrl si/veh; n = 115 cells for Gfat1 si/veh; n = 116 cells for ctrl si/TMG; n = 154 cells for Gfat1 si/TMG. At least 3 independent experiments were conducted with 2-3 samples/group/experiment. Data are shown as mean ± SEM. Significance was calculated by two-way ANOVA, followed by Tukey's test.

d. Treatments by GlcN, GlcNAc, and TMG led to increases in O-GlcNAcylation, respectively. N = 6-13. Data are shown as mean ± SEM. Student's t test (two-tailed) was conducted. ***, $p < 0.001$. Source data are provided as a Source Data file.
