# Supplementary material for: Chronic activation of hexosamine biosynthesis in the heart triggers pathological cardiac remodeling
Source: Nat Commun. 2020 Apr 14;11:1771. doi: 10.1038/s41467-020-15640-y (PMC7156663; doi:10.1038/s41467-020-15640-y)
Supplement: Supplementary file 2 — Reporting Summary [file 41467_2020_15640_MOESM2_ESM.pdf]

nature research

Corresponding author(s): Zhao Wang

Last updated by author(s): Feb 22, 2020

Reporting Summary

Nature Research wishes to improve the reproducibility of the work that we publish. This form provides structure for consistency and transparency in reporting. For further information on Nature Research policies, see Authors & Referees and the Editorial Policy Checklist.

Statistics

For all statistical analyses, confirm that the following items are present in the figure legend, table legend, main text, or Methods section.

n/a	Confirmed	
☐	☒	The exact sample size (n) for each experimental group/condition, given as a discrete number and unit of measurement
☐	☒	A statement on whether measurements were taken from distinct samples or whether the same sample was measured repeatedly
☐	☒	The statistical test(s) used AND whether they are one- or two-sided<br>*Only common tests should be described solely by name; describe more complex techniques in the Methods section.*
☐	☒	A description of all covariates tested
☐	☒	A description of any assumptions or corrections, such as tests of normality and adjustment for multiple comparisons
☐	☒	A full description of the statistical parameters including central tendency (e.g. means) or other basic estimates (e.g. regression coefficient) AND variation (e.g. standard deviation) or associated estimates of uncertainty (e.g. confidence intervals)
☐	☒	For null hypothesis testing, the test statistic (e.g. F, t, r) with confidence intervals, effect sizes, degrees of freedom and P value noted<br>*Give P values as exact values whenever suitable.*
☒	☐	For Bayesian analysis, information on the choice of priors and Markov chain Monte Carlo settings
☒	☐	For hierarchical and complex designs, identification of the appropriate level for tests and full reporting of outcomes
☒	☐	Estimates of effect sizes (e.g. Cohen's d, Pearson's r), indicating how they were calculated

Our web collection on statistics for biologists contains articles on many of the points above.

Software and code

Policy information about availability of computer code

Data collection	1) Roche LightCycler 480, software release 1.5.1.6.2 SP2, and Bio-Rad CFX96 (or 384) Realtime PCR system, CFX Maestro 1.1, for qPCR data acquisition.<br>2) Odyssey infrared imaging system, application software version 3.0, for Western blotting data collection.<br>3) VisualSonics, #Vevo 2100, MS400C probe, for echocardiography data acquisition.<br>4) Image J, 1.52P, for heart cardiomyocyte cross-sectional area and cultured cardiomyocyte cell surface area calculation.<br>5) Beckman scintillation counter, #LS5000TA, for radioactive 3H-leucine measurements.
Data analysis	Microsoft Excel 14.7.7. and Graphpad Prism 7.01, to generate graphs and conduct statistical analysis.

For manuscripts utilizing custom algorithms or software that are central to the research but not yet described in published literature, software must be made available to editors/reviewers. We strongly encourage code deposition in a community repository (e.g. GitHub). See the Nature Research guidelines for submitting code & software for further information.

Data

Policy information about availability of data

All manuscripts must include a data availability statement. This statement should provide the following information, where applicable:

- Accession codes, unique identifiers, or web links for publicly available datasets
- A list of figures that have associated raw data
- A description of any restrictions on data availability

All relevant data are available from the corresponding author upon reasonable request.

Field-specific reporting

Please select the one below that is the best fit for your research. If you are not sure, read the appropriate sections before making your selection.

☒ Life sciences ☐ Behavioural & social sciences ☐ Ecological, evolutionary & environmental sciences

For a reference copy of the document with all sections, see nature.com/documents/nr-reporting-summary-flat.pdf

Life sciences study design

All studies must disclose on these points even when the disclosure is negative.

Sample size	No statistical method was used to pre-determine sample size. All sample size information is provided in the figure legends.
Data exclusions	No data were excluded from analysis.
Replication	All attempts at replication were successful. Most graphs represent the collective data from multiple independent experiments and the number of replicates are given in figure legends.
Randomization	For mouse experiments, animals of the same litters were randomly assigned and compared.
Blinding	Investigators were blinded in the process of data collection, processing, and analysis.

Reporting for specific materials, systems and methods

We require information from authors about some types of materials, experimental systems and methods used in many studies. Here, indicate whether each material, system or method listed is relevant to your study. If you are not sure if a list item applies to your research, read the appropriate section before selecting a response.

Materials & experimental systems

n/a	Involved in the study	
☐	☒	Antibodies
☒	☐	Eukaryotic cell lines
☒	☐	Palaeontology
☐	☒	Animals and other organisms
☒	☐	Human research participants
☒	☐	Clinical data

Methods

n/a	Involved in the study	
☒	☐	ChIP-seq
☒	☐	Flow cytometry
☒	☐	MRI-based neuroimaging

Antibodies

Antibodies used

The following antibodies were used: Gfat1 (Santa Cruz Biotechnology, #sc-134894), GalE (Abcam, #ab155997), Gnpnat1 (Sigma, #HPA044647), Pgm3 (Sigma, #WH0005238M1), Uap1 (Sigma, #SAB1406469), GAPDH (Fitzgerald, #10R-G109A), p-Akt (Cell Signaling, #9271), Akt (Cell Signaling, #2920), mTOR (Cell Signaling, #4517), p-mTOR (Cell Signaling, #2974), S6 (Cell Signaling, #2217), p-S6 (Cell Signaling, #5364), 4EBP1 (Cell Signaling, #9644), p-4EBP1 (Cell Signaling, #2855), Anf (Abcam, #ab180649), βMHC (Abcam, #ab124205), RCAN1 (Sigma, #D6694), O-GlcNAc (ThermoFisher, MA1-072), IRDye 800 CW goat anti-rabbit secondary antibody (Li-Cor, #925-32211), and Alexa Fluor 700-conjugated goat anti-mouse secondary antibody (ThermoFisher, #A-21036). All primary antibodies were used at 1:1000. All secondary antibodies were used at 1:5000.

Validation

Gfat1 antibodies were validated by using Gfat1 overexpression and knockdown primary cells, and Gfat1 overexpression mouse hearts.
All other antibodies used in this study have also been validated. The information can be found on the manufacturer's websites as listed below.
GalE (Abcam, #ab155997, clone number EPR11088[B]), https://www.abcam.com/gale-antibody-epr11088b-ab155997.html
Gnpnat1 (Sigma, #HPA044647), https://www.sigmaaldrich.com/catalog/product/sigma/hpa044647?lang=en&region=US
Pgm3 (Sigma, #WH0005238M1, clone 1E2-1B12), https://www.sigmaaldrich.com/catalog/product/sigma/wh0005238m1?lang=en&region=US
Uap1 (Sigma, #SAB1406469), https://www.sigmaaldrich.com/catalog/product/sigma/sab1406469?lang=en&region=US
GAPDH (Fitzgerald, #10R-G109A, clone 6C5), https://www.fitzgerald-fii.com/gapdh-antibody-10r-g109a.html
p-Akt (Cell Signaling, #9271), https://www.cellsignal.com/products/primary-antibodies/phospho-akt-ser473-antibody/9271
Akt (Cell Signaling, #2920, clone 40D4), https://www.cellsignal.com/products/primary-antibodies/akt-pan-40d4-mouse-mab/2920
mTOR (Cell Signaling, #4517, clone L27D4), https://www.cellsignal.com/products/primary-antibodies/mtor-l27d4-mouse-mab/4517
p-mTOR (Cell Signaling, #2974), https://www.cellsignal.com/products/primary-antibodies/phospho-mtor-ser2481-antibody/2974
S6 (Cell Signaling, #2217, clone 5G10), https://www.cellsignal.com/products/primary-antibodies/s6-ribosomal-protein-5g10-

rabbit-mab/2217
p-S6 (Cell Signaling, #5364, clone D68F8), https://www.cellsignal.com/products/primary-antibodies/phospho-s6-ribosomal-protein-ser240-244-d68f8-xp-rabbit-mab/5364
4EBP1 (Cell Signaling, #9644, clone 53H11), https://www.cellsignal.com/products/primary-antibodies/4e-bp1-53h11-rabbit-mab/9644
p-4EBP1 (Cell Signaling, #2855, clone 236B4), https://www.cellsignal.com/products/primary-antibodies/phospho-4e-bp1-thr37-46-236b4-rabbit-mab/2855
Anf (Abcam, #ab180649), https://www.abcam.com/natriuretic-peptides-a-antibody-ab180649.html
βMHC (Abcam, #ab124205), https://www.abcam.com/heavy-chain-myosinmyh3-antibody-ab124205.html
RCAN1 (Sigma, #D6694), https://www.sigmaaldrich.com/catalog/product/sigma/d6694?lang=en&region=US
O-GlcNAc (ThermoFisher, MA1-072, clone RL2), https://www.thermofisher.com/antibody/product/O-linked-N-acetylglucosamine-O-GlcNAc-Antibody-clone-RL2-Monoclonal/MA1-072
IRDye 800 CW goat anti-rabbit secondary antibody (Li-Cor, #925-32211), https://www.licor.com/bio/reagents/irdye-800cw-goat-anti-rabbit-igg-secondary-antibody
Alexa Fluor 700-conjugated goat anti-mouse secondary antibody (ThermoFisher, #A-21036), https://www.thermofisher.com/antibody/product/Goat-anti-Mouse-IgG-H-L-Cross-Adsorbed-Secondary-Antibody-Polyclonal/A-21036

Animals and other organisms

Policy information about studies involving animals; ARRIVE guidelines recommended for reporting animal research

Laboratory animals	This study involved mice. The strain information (C57BL/6) is described in the Methods section. Male mice were used for most experiments. The age of mice for all experiments is either stated in the figure legends or in the Methods section.
Wild animals	No wild animals were used in this study.
Field-collected samples	No field-collected samples were used in this study.
Ethics oversight	All animal procedures have been approved by the Institutional Animal Care and Use Committee of University of Texas Southwestern Medical Center.

Note that full information on the approval of the study protocol must also be provided in the manuscript.
